# Specificity and genetic polymorphism in the Vfm quorum sensing system of plant pathogenic bacteria of the genus *Dickeya*

**DOI:** 10.1111/1462-2920.15889

**Published:** 2022-01-10

**Authors:** Nicole Hugouvieux‐Cotte‐Pattat, Monique Royer, Erwan Gueguen, Paul Le Guen, Roderich D. Süssmuth, Sylvie Reverchon, Stéphane Cociancich

**Affiliations:** ^1^ Univ Lyon, Université Claude Bernard Lyon 1, INSA‐Lyon, CNRS, UMR5240 MAP Villeurbanne F‐69622 France; ^2^ CIRAD, UMR PHIM Montpellier F‐34398 France; ^3^ PHIM, Univ Montpellier, CIRAD, INRAE, Institut Agro, IRD Montpellier France; ^4^ Institut für Chemie, Technische Universität Berlin Berlin D‐10623 Germany

## Abstract

The Vfm quorum sensing (QS) system is preponderant for the virulence of different species of the bacterial genus *Dickeya*. The *vfm* gene cluster encodes 26 genes involved in the production, sensing or transduction of the QS signal. To date, the Vfm QS signal has escaped detection by analytical chemistry methods. However, we report here a strain‐specific polymorphism in the biosynthesis genes *vfmO* and *vfmP*, which is predicted to be related to the production of different analogues of the QS signal. Consequently, the Vfm communication could be impossible between strains possessing different variants of the genes *vfmO/P*. We constructed three Vfm QS biosensor strains possessing different *vfmO/P* variants and compared these biosensors for their responses to samples prepared from 34 *Dickeya* strains possessing different *vfmO/P* variants. A pattern of specificity was demonstrated, providing evidence that the polymorphism in the genes *vfmO/P* determines the biosynthesis of different analogues of the QS signal. Unexpectedly, this *vfmO/P*‐dependent pattern of specificity is linked to a polymorphism in the ABC transporter gene *vfmG*, suggesting an adaptation of the putative permease VfmG to specifically bind different analogues of the QS signal. Accordingly, we discuss the possible involvement of VfmG as co‐sensor of the Vfm two‐component regulatory system.

## Introduction

Gram‐negative bacteria belonging to the genus *Dickeya* (formerly *Erwinia chrysanthemi*) are responsible for various crop diseases affecting many plant species of agronomic importance as well as ornamental plants (Charkowski *et al*., 2012; Hugouvieux‐Cotte‐Pattat *et al*., 2020a). Currently, 12 species have been characterized in the genus *Dickeya*: *D*. *aquatica*, *D*. *chrysanthemi*, *D*. *dadantii*, *D*. *dianthicola*, *D*. *fangzhongdai*, *D*. *lacustris*, *D*. *oryzae*, *D*. *parazeae*, *D*. *poaceiphila*, *D*. *solani*, *D*. *undicola* and *D*. *zeae* (Samson *et al*., 2005; Brady *et al*., 2012; van der Wolf *et al*., 2014; Tian *et al*., 2016; Hugouvieux‐Cotte‐Pattat *et al*., 2019; Oulghazi *et al*., 2019; Wang *et al*., 2020; Hugouvieux‐Cotte‐Pattat *et al*., 2020b; Hugouvieux‐Cotte‐Pattat and Van Gijsegem, 2021). The species *D*. *paradisiaca* was recently recognized as a new genus, *Musicola*, which includes two species *M*. *paradisiaca* and *M*. *keenii* (Hugouvieux‐Cotte‐Pattat *et al*., 2021). Strains from the genus *Dickeya* or *Musicola* belong to the order *Enterobacterales*, along with the genera *Brenneria*, *Lonsdalea*, *Pectobacterium* and *Sodalis* (Adeolu *et al*., 2016). *Dickeya* and *Pectobacterium* are plant pathogenic bacteria giving similar symptoms of soft‐rot on a large number of plants (Ma *et al*., 2007). They largely secrete pectate lyases to degrade the plant cell wall pectin, leading to the dissociation and maceration of the plant tissues (Hugouvieux‐Cotte‐Pattat *et al*., 2014). Of specific concern are three *Dickeya* species: *D*. *solani* provoking emergent damages in potato fields, particularly in Europe, resulting in its classification as a quarantine organism in Israel and Ireland (Toth *et al*., 2011), whereas *D*. *dianthicola* causes severe losses in potato production in the United States (Curland *et al*., 2021) and *D*. *oryzae* provokes significant losses in rice fields in China (Lv *et al*., 2019).

Quorum sensing (QS) is a cell‐to‐cell communication process that enables bacteria to collectively, and in a synchronized manner, modify their behaviour in response to changes in the cell density. Among the functions under QS control stand the formation of biofilms, the acquisition of nutrients, the conjugative transfer of plasmids, the production of antibiotics, and, more generally, the production of virulence factors (for reviews, see Grandclément *et al*., 2016; Hawver *et al*., 2016; Papenfort and Bassler, 2016). A successful bacterial infection is based on finely tuned regulatory mechanisms, including QS systems. Two QS mechanisms have been described in *Dickeya*. The first system is typically found in Gram‐negative bacteria and involves the production and the cytoplasmic recognition of *N*‐acyl‐homoserine lactones (Nasser *et al*., 1998). The second QS system is original, depending on a signal encoded by a locus called *vfm* for virulence factor modulating (Nasser *et al*., 2013). All 126 sequenced genomes of *Dickeya* strains available until April 2021 do contain the *vfm* locus but, so far, the Vfm QS system has been studied only in three species: it was shown to be preponderant for the virulence of the *D*. *dadantii* strain 3937 (Nasser *et al*., 2013) and for various strains of the agronomically important potato pathogen *D*. *solani* (Potrykus *et al*., 2018). In strain EC1, a rice pathogen recently reclassified as *D*. *oryzae* (Wang *et al*., 2020), the Vfm QS system also modulates multiple virulence traits (Lv *et al*., 2019).

**Fig. 1:**
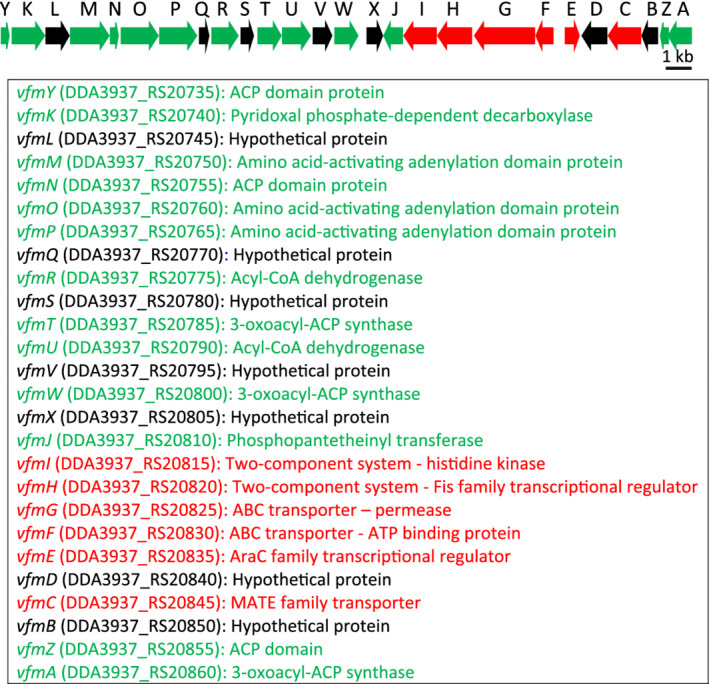
Functional annotation of the *vfm* gene cluster of *Dickeya dadantii* 3937 (taken from the GenBank accession NC**_**014500**).** In the upper part, the 26 genes of the gene cluster are presented by arrows. Green arrows correspond to genes annotated as biosynthesis genes, red arrows are for genes annotated as regulatory or transporter genes and black arrows for genes encoding hypothetical proteins. The length of arrows is proportional to the gene length according to the scale indicated at the right. The predicted function of each gene is shown below, with the same colour code. Abbreviations: ABC: ATP binding cassette, ACP: acyl carrier protein, MATE: multidrug and toxic compound extrusion. The corresponding GenBank tags are indicated for each *vfm* genes.

Initially, the *vfm* locus was discovered and genetically characterized in the model strain *D*. *dadantii* 3937 (Nasser *et al*., 2013). It encodes 26 genes annotated as *vfmA* to *vfmZ* and is expected to be required for the biosynthesis, sensing or transduction of the Vfm QS signal (Fig. 1). It does not share any gene homology with other known and studied QS loci, indicating that both the structure of the Vfm QS signal and its corresponding signalling cascade exhibit unique features. Despite several attempts, the Vfm QS signal has never been isolated and has escaped detection by analytical chemistry methods (Nasser *et al*., 2013; Lv *et al*., 2019). The products of the genes *vfmM*, *vfmO* and *vfmP* are annotated as amino acid‐activating adenylation domain proteins, indicating that the Vfm signal is a non‐ribosomally synthesized peptide. Short peptides used as QS signals are common among Gram‐positive bacteria, but these autoinducing peptides (AIP) correspond to ribosomally synthesized peptides. Each AIP is synthesized as a longer peptide precursor which is subsequently exported and modified (Otto *et al*., 1998; Cook and Federle, 2014).

Based on functional studies in *D*. *dadantii* 3937 and *D*. *oryzae* EC1, the *vfm* locus was shown to activate virulence through a signalling cascade involving the two‐component system VfmI/VfmH (Nasser *et al*., 2013; Lv *et al*., 2019). When the Vfm QS signal accumulates in the extracellular medium up to a certain threshold concentration, this two‐component system initiates the transcriptional expression of the *vfm* genes, rapidly provoking the activation of the expression of virulence genes (Nasser *et al*., 2013; Lv *et al*., 2019). Interestingly, the AIP QS system of several Gram‐positive bacteria also employs a two‐component regulatory system for the signal transduction and activation of the virulence genes (Hoch and Silhavy, 1995; Hvarstein, 2003). The most studied example is the Agr QS system of the genus *Staphylococcus*. A remarkable feature of the *agr* locus is the genetic link between a polymorphism observed within the ligand‐binding domain of the histidine kinase and a polymorphism observed within the sequence of the mature AIP signal (Dufour *et al*., 2002). In *Staphylococcus aureus*, these genetically linked allelic variations were shown to define four Agr QS specificity groups, each producing a unique AIP analogue associated with a unique sequence of the ligand‐binding domain of the histidine kinase. Among the four AIP analogue–histidine kinase pairs, two are more efficient for the transduction of the QS signal, illustrating the fact that the polymorphism observed in the *agr* locus of *S*. *aureus* affects the bacterial pathogenicity by impacting the efficiency of the Agr QS system (Geisinger *et al*., 2012).

A previous comparative genomic analysis of 47 strains of the genus *Dickeya* was performed to understand the phylogenetic relationship between the different species (Duprey *et al*., 2019). In this work, several phylogenetic trees were constructed both on core proteins and on proteins involved in the bacterial virulence. While the phylogenetic trees based on core proteins largely correlate with the species classification, we noticed that this correlation is much less clear for the phylogenetic trees of some Vfm proteins (Duprey *et al*., 2019). This result suggests that horizontal gene transfer occurred among the species of *Dickeya* at the level of some *vfm* genes. For example, the proteins VfmG and VfmP of the *D*. *dadantii* strain NCPPB 3537 belong to phylogenetic groups distant from those containing the proteins VfmG and VfmP of other *D*. *dadantii* strains. In all cases, however, the phylogenetic trees of VfmG and VfmP correlate with each other, suggesting a link between the polymorphism in the gene *vfmG* and the polymorphism in the gene *vfmP*. While the gene *vfmG* encodes the permease component of an ABC transporter, the gene *vfmP* encodes an amino acid‐activating adenylation domain protein predicted to govern the specific selection of one amino acid during the non‐ribosomal assembly of the peptidic part of the Vfm QS signal.

Some analogies with the genetically linked allelic variations observed within the *agr* locus, which define four QS specificity groups in *S*. *aureus*, raise several questions about the functioning of the *Dickeya* Vfm QS system. Is the polymorphism in the biosynthesis gene *vfmP* related to the production of different analogues of the QS signal? What is the functional link between the biosynthesis gene *vfmP* and the ABC permease gene *vfmG* that could explain the correlated polymorphism observed in these two genes? Is there polymorphism in other *vfm* genes, in addition to *vfmG* and *vfmP*? Does the polymorphism in the *vfm* genes modify the specificity of the Vfm QS system? In order to address these questions, we further analyzed the polymorphism in the *vfm* genes and we propose hypotheses regarding the impact of the polymorphism in the *vfm* genes on the specificity of the Vfm QS system. Finally, we obtained experimental proof using three newly constructed Vfm biosensor strains and a large set of *Dickeya* wild‐type strains that allowed us to explore and validate these hypotheses.

## Results

### Identification of polymorphism in VfmO and VfmP by prediction of the substrate specificity of the Vfm amino acid‐­activating adenylation domain proteins

The genes *vfmM*, *vfmO* and *vfmP* encode the three amino acid‐­activating adenylation domain proteins VfmM, VfmO and VfmP respectively. Such stand‐alone adenylation domain proteins are specific to the type II non‐ribosomal peptide synthesis (NRPS) (for review, Jaremko *et al*., 2020). In bacteria, type II NRPS has been less studied than type I NRPS which involves large modular and multidomain synthetases (for review, Süssmuth and Mainz, 2017). Nevertheless, we used the well‐documented data available on the bacterial type I NRPS to predict the nature of the amino acids activated by the proteins VfmM, VfmO and VfmP, respectively. The models of prediction of the bacterial type I NRPS are based on a substrate specificity‐conferring signature corresponding to 10 residues present at identical positions in the amino acid sequence of all type I NRPS adenylation domains (Stachelhaus *et al*., 1999; Finking and Marahiel, 2004; Süssmuth and Mainz, 2017). The 10 residues of these signatures are identified on the basis of the alignment of the amino acid sequence of the protein to be characterized versus the amino acid sequence of experimentally characterized adenylation domains. We used these models to analyze the potential polymorphism of the proteins VfmM, VfmO and VfmP among the 126 *Dickeya* strains whose genome sequences are available in public databases until April 2021 (Table 1). The protein VfmM is predicted to incorporate the same amino acid in the 126 sequenced *Dickeya* strains, as the proteins VfmM of all these strains exhibit the same type I NRPS‐like substrate specificity‐conferring signature (data not shown). In contrast, different forms of the proteins VfmO and VfmP, resulting from genetic polymorphism, are predicted to incorporate different amino acids according to the strains. We identified three and four different type I NRPS‐like substrate specificity‐conferring signatures for the proteins VfmO and VfmP respectively (Table 1). The position in the sequences of the proteins VfmO and VfmP of the residues identified as constituting these signatures is provided in Fig. S1. To further analyze these signatures, we used the same alignment to also determine the type I NRPS‐like substrate specificity‐conferring signature of the type II NRPS stand‐alone adenylation domain protein DltA, which is required for the biosynthesis of cell wall in *Bacillus subtilis* and which was experimentally shown to be specific for d‐alanine (Kittilä *et al*., 2016). Interestingly, this DltA signature (**D**LMT**MCTVAK**) shares six or seven residues with the three signatures identified for the protein VfmO (**D**AYVI**CTVAK**, **D**VYVI**CTVAK**or **D**VFV**MCTVAK**; Table 1). However, since none of the VfmO signatures matches exactly to the DltA signature, it is not possible to predict that one of them is specific for d‐alanine.

**Table 1:** Distribution into five Vfm genetic groups of strains belonging to different species of *Dickeya*.

Vfm genetic groups	Strains representative of the 12 *Dickeya* species^a^	NRPS substrate specificity‐conferring signatures associated to each variant of the genes *vfmO* and *vfmP* ^b^		Combinations of variants of the genes *vfmO* and *vfmP*	Number of strains identified in each Vfm genetic group^c^
I	*D*. *dadantii* DSM 18020^T^; *D*. *dadantii* 3937; *D*. *dianthicola* NCPPB 453^T^; ***D*. *fangzhongdai* NCPPB 2929**	*vfmO1* D**A**YVICTVAK	*vfmP1* DMMFF**A**ACVK	*vfmO1/P1*	38
II	*D*. *oryzae* EC2; *D*. *chrysanthemi* NCPPB 516 (CFBP 1270)		*vfmP2* DMMFFGACVK	*vfmO1/P2*	3
III	*D*. *chrysanthemi* NCPPB 3533; *D*. *fangzhongdai* DSM 101947^T^; *D*. *oryzae* ZYY5^T^; *D*. *oryzae* EC1; *D*. *parazeae* S31^T^; *D*. *solani* IPO 2222^T^; *D*. *undicola* FVG1‐MFK‐O17; *D*. *zeae* MS1	*vfmO2* DVYVICTVAK		*vfmO2/P2*	61
IV	*D*. *aquatica* 174/2^T^; *D*. *chrysanthemi* NCPPB 402^T^; ***D*. *dadantii* NCPPB 3065; *D*. *dadantii* CFBP 3964;** *D*. *fangzhongdai* S1; *D*. *lacustris* S29^T^; *D*. *parazeae* Ech586; *D*. *solani* RNS 05.1.2A; *D*. *undicola* 2B12^T^; *D*. *zeae* NCPPB 2538^T^		*vfmP3* D**L**MF**A**G**C**C**M**K	*vfmO2/P3*	25
V	*D*. *poaceiphila* NCPPB 569^T^; *D*. *poaceiphila* CFBP 2040	*vfmO3* DV**F**V**M**CTVAK	*vfmP4* DM**L**FFGAC**L**K	*vfmO3/P4*	2

^a^ For each species, the table provides data for the type strain and for at least one example of strain identified as belonging to each Vfm group. The absence of a species in a Vfm genetic group indicates that no strain of this species has been identified in this group.

^b^ Residues differing from the substrate specificity‐conferring signatures VfmP and VfmO of *D*. *solani* IPO 2222^T^ (group III) are written in bold letters.

^c^ This analysis includes the 126 strains of *Dickeya* whose genomes are available until April 2021 plus the three strains whose genomes have been sequenced in the frame of this study (*D*. *fangzhongdai* NCPPB 2929, *D*. *dadantii* NCPPB 3065 and *D*. *dadantii* CFBP 3964, shown in bold letters).

Since none of the three VfmO signatures and none of the four VfmP signatures match exactly to any known type I NRPS‐like specificity‐conferring signature or to the DltA signature, predictions regarding the nature of the activated amino acid substrate have not been possible. Nevertheless, the variations in the signatures of VfmO and VfmP suggest the production of different analogues of the Vfm QS signal, each analogue differing at the level of the amino acids activated by the enzymes VfmO or VfmP. Accordingly, different combinations of the variants of the genes *vfmO* and *vfmP* are predicted to be related to the production of different analogues of the Vfm QS signal. Among the 126 sequenced *Dickeya* strains, the three variants of the gene *vfmO* associated with different NRPS signatures have been referred to as *vfmO1* to *vfmO3*, and the four variants of the gene *vfmP* associated with different NRPS signatures have been referred to as *vfmP1* to *vfmP4*, respectively (Table 1, Fig. S1).

### The *Dickeya* strains are split into five *vfmO/P* genetic groups predicted to produce different analogues of the Vfm QS signal

Five different combinations of the variants resulting from the *vfmO* and *vfmP* polymorphism have been identified among the 126 sequenced *Dickeya* strains (Table 1). Thus, five Vfm genetic groups designated as groups I to V have been referred to as strains possessing the combinations *vfmO1*/*P1*, *vfmO1/P2*, *vfmO2/P2*, *vfmO2/P3* and *vfmO3/P4*, respectively.

**Fig. 2:**
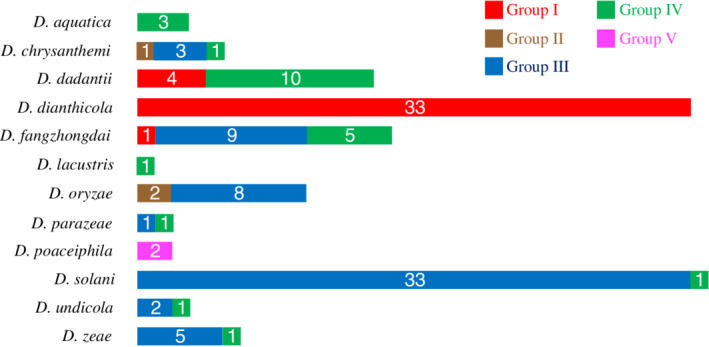
Distribution of strains of the five Vfm genetic groups within the 12 species of *Dickeya*. The length of each block is proportional to the number of strains indicated in the block. The colour of the blocks refers to the Vfm genetic group as indicated at the right of the figure. These data are based on the analysis of the 126 *Dickeya* genomic sequences available in GenBank until April 2021 plus the genomic sequences of the strains *D*. *fangzhongdai* NCPPB 2929, *D*. *dadantii* NCPPB 3065 and *D*. *dadantii* CFBP 3964 obtained in the present study.

Among them, the groups I, III and IV include the majority of the sequenced strains (38, 61 and 25 respectively) while the groups II or V contain only three and two strains, respectively (Table 1). Interestingly, the strain appurtenance to a Vfm genetic group is not correlated with the strain classification into species (Table 1; Fig. 2). Most species include strains that are distributed into two or three different Vfm genetic groups, as is the case for *D*. *chrysanthemi*, *D*. *dadantii*, *D*. *fangzhongdai*, *D*. *oryzae*, *D*. *parazeae*, *D*. *undicola* and *D*. *zeae* (Fig. 2; Table S1). In contrast, four species seem to be mostly or solely associated with one group. All the 33 *D*. *dianthicola* strains belong to group I and all but one of the 34 *D*. *solani* strains are members of group III (Fig. 2, Table S1). The four *D*. *aquatica* or *D*. *lacustris* strains belong to group IV, and the two *D*. *poaceiphila* strains belong to group V (Fig. 2; Table S1).

The five Vfm genetic groups, defined by the polymorphism of the NRPS substrate specificity‐conferring signatures of the proteins VfmO and VfmP, are expected to perform the biosynthesis of different analogues of the Vfm QS signal. We attempted to explore this hypothesis with experimental data.

### Analysis of 52 *Dickeya* strains for their capacity to activate the Vfm *uidA* biosensor strain (group I)

A Vfm biosensor strain was previously constructed for the *D*. *dadantii* strain 3937 (Nasser *et al*., 2013) which belongs to the Vfm genetic group I (Table 1). This strain, called biosensor I‐uidA in the present study, corresponds to an insertion knock‐out mutant of the gene *vfmE* in which the promoter of this gene is fused to the reporter gene *uidA* encoding β‐glucuronidase (Nasser *et al*., 2013). In order to study the ability of the biosensor I‐uidA to detect and respond to the Vfm QS signal produced by *Dickeya* strains belonging to different Vfm genetic groups, we tested cell‐free supernatants of liquid cultures of a collection of 52 strains representative of the different *Dickeya* species (Table 2). Since the Vfm genetic group of strains whose genomic sequences are available was previously determined (Table S1), the group appurtenance of 31 tested strains was known. A positive response was observed for all sequenced strains belonging to groups I or II (six and one strains, respectively, Table 2). In contrast, a negative response was observed with all sequenced strains belonging to any of the other three groups (14, eight and two strains for groups III, IV and V, respectively) (Table 2). To confirm the correlation observed between the positive response to the biosensor I‐uidA and the appurtenance to groups I or II, we sequenced the genomes of the *D*. *fangzhongdai* strain NCPPB 2929 which gave a positive response and of two *D*. *dadantii* strains, NCPPB 3065 and CFBP 3694, which gave a negative response (Table 2). We also re‐sequenced the genome of the positive strain *D*. *chrysanthemi* strain CFBP 1270 (NCPPB 516) because this strain was the only experimentally tested strain of group II. Furthermore, the genomic sequence of this strain available in GenBank contains a frameshift in the *vfmO* gene. Analysis of the resulting sequences confirmed that *D*. *chrysanthemi* CFBP 1270 belongs to group II and shows that there is no frameshift in the *vfmO* gene of this strain. Regarding the three newly sequenced strains, *D*. *fangzhongdai* NCPPB 2929 belongs to group I, and both *D*. *dadantii* strains NCPPB 3065 and CFBP 3694 belong to group IV. These additional genomic data are in full agreement with the response observed for these three strains, also showing that only strains belonging to groups I or II give a positive response to the biosensor I‐uidA.

**Table 2:** Response of the Vfm biosensor I‐uidA to cell‐free supernatants of cultures of *Dickeya* strains belonging to different species and to different Vfm genetic groups.

Species^a^	Strains	Groups^b^	Response^c^	Species^a^	Strains	Groups^b^	Response^c^
*D*. *aquatica*	174/2^T^ (NCPPB 4580^T^)	IV	0/5	*D*. *lacustris*	S39 (CFBP 8649)	n/a	0/5
	DW 0440	IV	0/5		J114b (CFBP 8721)	n/a	0/5
	FL37	n/a	0/5	*D*. *oryzae*	NCPPB 3531 (CFBP 8729)	III	0/5
	JDA74 (CFBP 8722)	n/a	0/5		CFBP 8738 (DZ2Q)	III	0/5
*D*. *chrysanthemi*	CFBP 2048^T^ (NCPPB 402^T^)	IV	0/5		CFBP 1271	n/a	0/5
	NCPPB 3533	III	0/5		CFBP 4148	n/a	0/5
	CFBP 1270 (NCPPB 516)	II	5/5		NCPPB 2547	n/a	0/5
	EC16 (ATCC 11662)	n/a	0/5	*D*. *poaceiphila*	NCPPB 569^T^ (CFBP 8731^T^)	V	0/5
	CFBP1346	n/a	0/5		CFBP 2040	V	0/5
*D*. *dadantii*	3937 (CFBP 3855)	I	5/5		CFBP 1537	n/a	0/5
	CFBP 1269^T^ (DSM 18020^T^)	I	5/5	*D*. *solani*	IPO 2222^T^ (NCPPB 4479^T^)	III	0/5
	CFBP 2051 (NCPPB 2976)	I	5/5		Ds0432‐1	III	0/5
	NCPPB 3537	IV	0/5		GBBC 2040	III	0/5
	NCPPB 3065 (ENA49)	IV	0/5		PPO 9019	III	0/5
	CFBP 3964	IV	0/5		RNS 08.23.3.1.A	III	0/5
*D*. *dianthicola*	RNS 04.9	I	5/5		RNS 05.1.2A	IV	0/5
	NCPPB 2421	n/a	5/5		CFBP 5647	n/a	0/5
	CFBP 1888 (NCPPB 3344)	I	5/5	*D*. *undicola*	2B12 (CFBP 8650)	IV	0/5
	CFBP 2982	I	5/5		FVG10‐MFV‐A16	III	0/5
*D*. *fangzhongdai*	NCPPB 3274	III	0/5		FVG1‐MFK‐O17	III	0/5
	DSM 101947^T^ (CFBP 8607 ^T^)	III	0/5		CFBP 7083	n/a	0/5
	B16 (CFBP 8496)	III	0/5	*D*. *zeae*	CFBP 2052^T^ (NCPPB 2538 ^T^)	IV	0/5
	NCPPB 2929	I	5/5		NCPPB 3532	III	0/5
	CFBP 1279 (NCPPB 1790)	n/a	0/5		CFBP 1268	n/a	0/5
	CFBP 6589	n/a	0/5		CFBP 1596	n/a	0/5
*D*. *lacustris*	S29^T^ (CFBP 8647^T^)	IV	0/5		CFBP 7084	n/a	0/5

^a^ Identification of the strains and their species appurtenance was verified by sequencing the *gapA* PCR product.

^b^ The Vfm genetic group has been determined for strains whose genome sequences are available in GenBank (Table S1) and for the three strains *D*. *fangzhongdai* NCPPB 2929 and *D*. *dadantii* NCPPB 3065 and CFBP 3964 whose genomes were sequenced in the frame of this study. n/a: Vfm genetic group not available.

^c^ Number of positive tests out of the total number of tests performed with the Vfm biosensor I‐uidA. Responses with positive tests are shown with a grey background.

### Comparison of the Vfm QS activity of 34 sequenced *Dickeya* strains using Vfm luciferase biosensor strains belonging to the three major groups I, III and IV, respectively

A Vfm luciferase biosensor for group I was constructed by introducing a plasmid harbouring a luciferase gene under the control of the promoter of the gene *vfmE* in the *D*. *dadantii* 3937 *vfmE* mutant. The resulting strain was called biosensor I‐luc. To construct Vfm luciferase biosensors corresponding to groups III and IV, we choose the *D*. *solani* strains IPO 2222 and RNS05‐1‐2A, respectively. In both strains, the *vfmE* coding sequence was deleted and replaced by the luciferase gene expressed under the control of the *vfmE* promoter. The resulting strains were called biosensors III‐luc and IV‐luc, respectively. The three luciferase biosensors I‐luc, III‐luc and IV‐luc were used to test cell‐free supernatants of liquid cultures of 34 sequenced strains of *Dickeya* previously tested with the biosensor I‐uidA. Regarding the biosensor I‐luc, a positive response was observed with all tested strains of groups I or II, and a negative response was observed with all tested strains belonging to any of the three other groups III, IV or V (Table 3). Regarding the biosensor IV‐luc, a positive response was observed with all tested strains of group IV, and a negative response was observed with all tested strains belonging to any of the four other groups (I, II, III or V) (Table 3). Regarding the biosensor III‐luc, a positive response was observed with the 14 strains of group III, and a negative response was observed with the 10 strains of group IV (Table 3). However, less reproducible positive responses of the biosensor III‐luc (one to four with five repeats) were also observed for six out of the seven strains of group I, the sole strain of group II and one of both strains of group V (Table 3). These assays have been repeated three times for the biosensors I‐luc and IV‐luc and five times for the biosensor III‐luc. Variations among repeats were observed when using the biosensor III‐luc with the cell‐free supernatants of several strains belonging to the groups I, II, or V and even with one strain of group III (Table 3).

**Table 3:** Response of the Vfm biosensors I‐luc, III‐luc and IV‐luc to cell‐free supernatants of cultures of *Dickeya* strains belonging to different Vfm genetic groups I to V.

Groups^a^	Strains	Response of the Vfm biosensors^b^		
		I‐luc	III‐luc	IV‐luc
I	*D*. *dadantii* CFBP 1269^T^ (DSM 18020^T^)	3/3	3/5	0/3
	*D*. *dadantii* CFBP 2051 (NCPPB 2976)	3/3	3/5	0/3
	*D*. *dadantii* 3937 (CFBP 3855)	3/3	1/5	0/3
	*D*. *dianthicola* RNS 04.9	3/3	2/5	0/3
	*D*. *dianthicola* CFBP 1888 (NCPPB 3344)	3/3	2/5	0/3
	*D*. *dianthicola* CFBP 2982	3/3	0/5	0/3
	*D*. *fangzhongdai* NCPPB 2929	3/3	3/5	0/3
II	*D*. *chrysanthemi* CFBP 1270 (NCPPB 516)	3/3	4/5	0/3
III	*D*. *chrysanthemi* NCPPB 3533	0/3	3/5	0/3
	*D*. *fangzhongdai* NCPPB 3274	0/3	5/5	0/3
	*D*. *fangzhongdai* DSM 101947^T^ (CFBP 8607 ^T^)	0/3	5/5	0/3
	*D*. *fangzhongdai* B16 (CFBP 8496)	0/3	5/5	0/3
	*D*. *oryzae* NCPPB 3531 (CFBP 8729)	0/3	5/5	0/3
	*D*. *oryzae* CFBP 8738 (DZ2Q)	0/3	5/5	0/3
	*D*. *solani* Ds0432‐1	0/3	5/5	0/3
	*D*. *solani* GBBC 2040	0/3	5/5	0/3
	*D*. *solani* PPO 9019	0/3	5/5	0/3
	*D*. *solani* RNS 08.23.3.1.A	0/3	5/5	0/3
	*D*. *solani* IPO 2222^T^ (NCPPB 4479^T^)	0/3	5/5	0/3
	*D*. *undicola* FVG10‐MFV‐A16	0/3	5/5	0/3
	*D*. *undicola* FVG1‐MFK‐O17	0/3	5/5	0/3
	*D*. *zeae* NCPPB 3532	0/3	5/5	0/3
IV	*D*. *aquatica* 174/2^T^ (NCPPB 4580^T^)	0/3	0/5	3/3
	*D*. *aquatica* DW 0440	0/3	0/5	3/3
	*D*. *chrysanthemi* CFBP 2048^T^ (NCPPB 402^T^)	0/3	0/5	3/3
	*D*. *dadantii* NCPPB 3065 (ENA49)	0/3	0/5	3/3
	*D*. *dadantii* CFBP 3964	0/3	0/5	3/3
	*D*. *dadantii* NCPPB 3537	0/3	0/5	3/3
	*D*. *lacustris* CFBP 8647^T^	0/3	0/5	3/3
	*D*. *solani* RNS 05.1.2A	0/3	0/5	3/3
	*D*. *undicola* 2B12 (CFBP 8650)	0/3	0/5	3/3
	*D*. *zeae* CFBP 2052^T^ (NCPPB 2538 ^T^)	0/3	0/5	3/3
V	*D*. *poaceiphila* CFBP 2040	0/3	4/5	0/3
	*D*. *poaceiphila* NCPPB 569^T^ (CFBP 8731^T^)	0/3	0/5	0/3

^a^ The Vfm genetic group has been determined for strains whose genome sequences are available in GenBank (Table S1) and also for the *D*. *fangzhongdai* strain NCPPB 2929 and the *D*. *dadantii* strains NCPPB 3065 and CFBP 3964 whose genomes were sequenced in the frame of this study.

^b^ Number of positive tests out of the total number of tests performed with the corresponding Vfm biosensor. Responses with at least one positive test are shown with a grey background.

### Comparison of the whole *vfm* gene cluster sequence from strains belonging to the same species but to different Vfm genetic groups

**Fig. 3:**
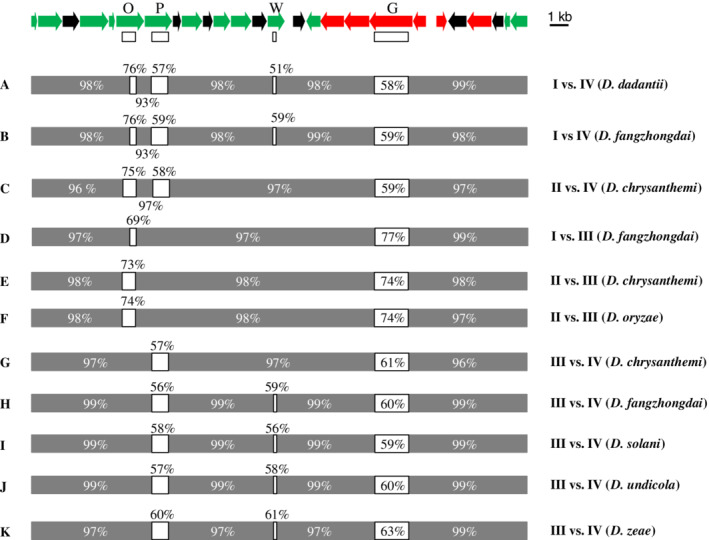
Characterization of variable regions in the *vfm* gene cluster between strains of *Dickeya* belonging to the same species but to different Vfm genetic groups. Top: Map of the *vfm* gene cluster. Genes are represented by arrows whose length is proportional to the gene length according to the scale indicated at the right. The genes *vfmO*, *vfmP*, *vfmW* and *vfmG* are indicated by the corresponding letter. White boxes below each of these four genes indicate the positions of the nucleotide sequences used to build the phylogenetic trees described in Fig. 4. A–K. Schematic representation of the alignment of nucleotide sequences of the *vfm* gene clusters for pairs of strains belonging to the same species but to different Vfm genetic groups (I, II, III or IV). Grey boxes correspond to the regions in which the aligned sequences of strain pairs share a nucleotide identity higher than 93%. White boxes correspond to the predicted recombined regions in which both aligned sequences share a nucleotide identity lower than 77%. Values of nucleotide identities shared by each strain pairs are indicated inside, below or above each box. A. *D*. *dadantii* 3937 (group I) versus *D*. *dadantii* NCPPB 3537 (group IV). B. *D*. *fangzhongdai* NCPPB 2929 (group I) versus *D*. *fangzhongdai* S1 (group IV). C. *D*. *chrysanthemi* NCPPB 516 (group II) versus *D*. *chrysanthemi* NCPPB 402T (group IV). D. *D*. *fangzhongdai* NCPPB 2929 (group I) versus *D*. *fangzhongdai* DSM 101947T (group III). E. *D*. *chrysanthemi* NCPPB 516 (group II) versus *D*. *chrysanthemi* NCPPB 3533 (group III). F. *D*. *oryzae* EC2 (group II) versus *D*. *oryzae* EC1 (group III). G. *D*. *chrysanthemi* NCPPB 3533 (group III) versus *D*. *chrysanthemi* NCPPB 402T (group IV). H. *D*. *fangzhongdai* DSM 101947T (group III) versus *D*. *fangzhongdai* S1 (group IV). I. *D*. *solani* IPO 2222T (group III) versus *D*. *solani* RNS 05.1.2A (group IV). J. *D*. *undicola* FVG1‐MFK‐O17 (group III) versus *D*. *undicola* 2B12T (group IV). K. *D*. *zeae* MS1 (group III) versus *D*. *zeae* NCPPB 2538T (group IV).

To identify other potential polymorphic genes associated with variation in the specificity of the Vfm QS system, we compared the nucleotide sequence of the *vfm* locus of strains belonging to the same species but to different Vfm genetic groups (groups I to V as defined by variations observed in the genes *vfmO* and *vfmP*). For example, alignment of the complete sequence of the *vfm* locus of the *D*. *dadantii* strain 3937 (group I) with the *D*. *dadantii* strain NCPPB 3537 (group IV) led to the identification of four polymorphic regions with low nucleotide identities (51%–76%) compared to the rest of the locus (93%–99%) (Fig. 3A). These four variable regions are located in the genes *vfmO*, *vfmP*, *vfmW* and *vfmG*, respectively. The comparative analysis of other pairs of strains belonging to the same species but to different Vfm genetic groups shows that recombination events occurred in the four genes *vfmO*, *vfmP*, *vfmW* and *vfmG* in *D*. *fangzhongdai* (pair I/IV, Fig. 3B), in the three genes *vfmO*, *vfmP* and *vfmG* in *D*. *chrysanthemi* (pair II/IV, Fig. 3C), in both genes *vfmO* and *vfmG* in *D*. *chrysanthemi*, *D*. *fangzhongdai* and *D*. *oryzae* (pairs I/III and II/III, Fig. 3D–F), in both genes *vfmP* and *vfmG* in *D*. *chrysanthemi* (pair III/IV, Fig. 3G) and in the three genes *vfmP*, *vfmW* and *vfmG* in *D*. *fangzhongdai*, *D*. *solani*, *D*. *undicola* and *D*. *zeae* (pairs III/IV, Fig. 3H–K). Thus, recombination events are frequently observed in the four genes *vfmO*, *vfmP*, *vfmW* and *vfmG* in at least seven *Dickeya* species. According to these genomic comparisons, the polymorphism already observed in the biosynthesis genes *vfmO* and *vfmP* appears to be associated with the polymorphism in the ABC permease gene *vfmG* and the oxoacyl‐ACP synthase gene *vfmW*.

**Fig. 4:**
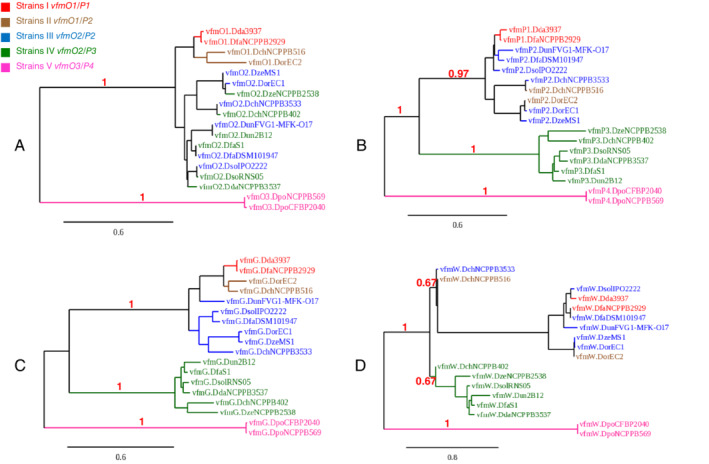
Phylogenetic trees of the variable regions of the genes *vfmO*, *vfmP*, *vfmG* and *vfmW* of a selection of representative *Dickeya* strains. A. *vfmO* tree. B. *vfmP* tree. C. *vfmG* tree. D. *vfmW* tree. The 18 selected strains correspond to triplets or pairs of strains, each triplet or pair belonging to the same species but to different Vfm genetic groups, except for *D*. *poaceiphila* whose two strains belong to group V. The colour code referring to their Vfm genetic group (strains I to V) is given at the top left corner. To simplify the figure, the names of the species are indicated as follows: Dch, *D*. *chrysanthemi* (three strains); Dda, *D*. *dadantii* (two strains); Dfa, *D*. *fangzhongdai* (three strains); Dor, *D*. *oryzae* (two strains); Dpo, *D*. *poaceiphila* (two strains); Dso, *D*. *solani* (two strains); Dun, *D*. *undicola* (two strains); Dze, *D*. *zeae* (two strains). The nucleotide sequences corresponding to the variable regions of the genes *vfmO* (727 nt), *vfmP* (901 nt), *vfmG* (2149 nt) and *vfmW* (229 nt) have been obtained from genome sequences available in GenBank (accession numbers given in Table S1), except for the strain NCPPB 2929 sequenced in the present study. The positions of these nucleotide sequences in the *vfm* gene cluster are indicated in Fig. 3. Nucleotide sequences have been aligned with MUSCLE (Edgar, 2004), the phylogenetic tree was reconstructed using the maximum likelihood method implemented in the PhyML program (Guindon and Gascuel, 2003) and graphical representation of the phylogenetic tree was performed with TreeDyn (Chevenet *et al*., 2006). Numbers at nodes correspond to bootstrap values (500 replicates of the original alignment). Bootstrap values are indicated only for the nodes that separate the different variants of the genes *vfmO* or *vfmP* or the main phyla for the genes *vfmG* or *vfmW*. The scale bar represents the average number of substitutions per site.

In order to obtain further insights into the potential links between the polymorphism in the genes *vfmO*, *vfmP*, *vfmG* and *vfmW*, phylogenetic trees were constructed using the nucleotide sequences of the variable regions identified in the genes *vfmO*, *vfmP*, *vfmG* and *vfmW* (Fig. 4). For this phylogenetic analysis, we selected 18 representative strains corresponding to respectively, two triplets and five pairs of strains belonging to the same species but to different Vfm genetic groups (I, II, III or IV) and two strains of group V. In the four resulting trees, the sequences of the two strains of group V are clearly separated in a specific phylum, indicating a more distant origin. The sequences of each *vfmO* variant (*vfmO1*, *vfmO2* and *vfmO3*) do cluster in a specific phylum, the variants *vfmO1* (groups I and II) and *vfmO2* (groups III and IV) being close to each other but well separated from the variants *vfmO3* (group V) (Fig. 4A). Similarly, the sequences of each *vfmP* variant (*vfmP1*, *vfmP2*, *vfmP3* and *vfmP4*) do cluster in a specific phylum, and the variants *vfmP1* (group I) and *vfmP2* (groups II and III) are close to each other but clearly separated from the two phyla formed by variants *vfmP3* (group IV) and *vfmP4* (group V) respectively (Fig. 4B). In the *vfmG* tree, the sequences of groups I, II and III are also close together but well separated from the two phyla formed by sequences of groups IV and V respectively (Fig. 4C). In the *vfmW* tree, the sequences of groups I, II and III cluster in a mixed phylum which is distinct from the two phyla formed by the sequences of groups IV and V, respectively (Fig. 4D).

## Discussion

### Biological validation of the prediction that the polymorphism in the genes *vfmO* and *vfmP* determines the production of different analogues of the Vfm QS signal

Based on an *in silico* analysis of the polymorphism of the *vfm* locus, the 126 sequenced strains of *Dickeya* can be classified into five Vfm genetic groups, each group possessing the same combination of variants of the biosynthesis genes *vfmO/vfmP* (Table 1). Strains belonging to the groups I to V will be referred to as strains I to V respectively. In order to explore the hypothesis that each Vfm genetic group produces a different analogue of the Vfm QS signal, bioassays have been performed. The latter are based on the design and generation of four biosensor strains belonging to the three major Vfm genetic groups (I, III and IV) Accordingly, the designation ‘analogues AI to AV’ will be used to refer to putative analogues of the Vfm QS signal produced by the five Vfm genetic groups respectively.

The two biosensors of group I, I‐uidA and I‐luc, both gave a positive response only with samples prepared from any of the tested strains of group I or II (Tables 2 and 3). This specificity pattern of strains I and II indicates that analogues AI and AII correspond to an identical or, with regard to the structure, at least closely related molecule(s) which differ(s) significantly from the signalling molecules produced by strains III, IV and V. The samples prepared from strains III gave a positive response with the biosensor III‐luc but not with the biosensors I‐uidA, I‐luc and IV‐luc (Table 3), indicating that the signalling molecules of type AIII differ from the analogues AI/AII and AIV. The biosensor of group IV, IV‐luc, gave a positive response only with samples prepared from any of the tested strains IV (Table 3). Additionally, the samples prepared from strains IV gave a positive response with the biosensor IV‐luc but not with the biosensors I‐uidA, I‐luc and III‐luc (Tables 2 and 3). This specificity pattern of strains from group IV suggests that the analogue AIV structurally differs from the analogues produced by strains I, II, III and V.

Altogether these results strongly suggest that strains I to IV produce at least three different signalling molecules of the Vfm QS signal (AI/AII, AIII and AIV), since only strains I and II seem to produce the same analogue. The isolation of the Vfm QS signals produced by strains belonging to different Vfm genetic groups, as well as the determination of their molecular mass and their chemical structure, will be required to definitively validate this hypothesis. Unfortunately, despite several attempts, the Vfm QS signal has not yet been isolated from any *Dickeya* strain (Nasser *et al*., 2013; Lv *et al*., 2019).

Regarding strains V, which include only two *D*. *poaceiphila* strains, the samples prepared from one strain gave a positive response with the biosensor III‐luc (Table 2), but the samples prepared from the second strain gave no positive response with the biosensors of groups I, III or IV. Thus, a larger set of strains V and a biosensor strain corresponding to group V would be required to more precisely determine the sensing specificity of this group.

### The NRPS substrate specificity‐conferring signatures associated with the different variants of the genes *vfmO* and *vfmP* are consistent with the production of different analogues of the Vfm QS signal

*In silico* analyses were performed to specify the non‐ribosomal code of 10 amino acids corresponding to the type I NRPS substrate specificity‐conferring signatures. The polymorphism in the biosynthesis genes *vfmO* and *vfmP* predict the incorporation of different amino acids in the Vfm QS signal, which gives rise to the production of different analogues of the Vfm QS signal differing by the amino acid sequence. This prediction is supported by the biological data obtained using the biosensor strains, which strongly suggest that the four genetic groups I to IV produce three distinct analogues of the Vfm QS signal.

The biological assays indicate that strains I and II synthesize functionally similar signals, suggesting that both combinations *vfmO1*/*P1* and *vfmO1*/*P2* result in the production of the same (or of a very similar) analogue. We thus compared the non‐ribosomal code of 10 amino acids corresponding to the VfmP1 and VfmP2 signatures: they differ by one amino acid located in position 6 (Table 1), a position which has been shown to be quite variable among type I NRPS signatures that specify the same amino acid (Stachelhaus *et al*., 1999; Eppelmann *et al*., 2002). This position of the signature almost always exhibits an aliphatic residue, either Ala or Gly which, indeed, are found at this position in the signatures associated with the variants *vfmP1* and *vfmP2*, respectively. Moreover, the sequences of the variants *vfmP1* and *vfmP2* are close in the *vfmP* phylogenetic tree (Fig. 4B), supporting the high level of similarity between these two variants. These observations suggest that the NRPS signatures associated with the variants *vfmP1* and *vfmP2* most probably confer the same amino acid specificity, leading to the biosynthesis of the same analogue of the Vfm signal in strains I and II.

The biological experiments indicate that strains I/II and III produce functionally different analogues, suggesting that the combinations *vfmO1*/*P1* or *vfmO1*/*P2* (strains I/II) are linked to the production of an analogue different of those produced by the combination *vfmO2*/*P2* (strains III). Interestingly, the VfmO1 and VfmO2 signatures also differ by only one amino acid (Fig. 2). However, this variable amino acid in position 2 of the signature is located in the entrance of the substrate‐specificity pocket and, as such, it plays a key role in the interaction between the type I NRPS adenylation domains and their substrate (Kudo *et al*., 2019). Accordingly, the NRPS signatures associated with the variants *vfmO1* and *vfmO2* are predicted to confer different substrate specificities, leading to the incorporation of different amino acids into the Vfm signal.

The NRPS signatures associated with the variants *vfmP2*, *vfmP3* and *vfmP4* differ by two to four residues (Table 1), most of them in positions where an enormous variability plays a major role in determining the substrate specificity (Stachelhaus *et al*., 1999; Eppelmann *et al*., 2002). This strongly supports the hypothesis that these three VfmP signatures confer specificity to three different amino acids, respectively. A similar observation was made for the NRPS signatures associated with the variants *vfmO2* and *vfmO3*, differing from the others by two out of 10 residues, and which are predicted to confer specificity to two different amino acids (Table 1). These *in silico* data support the hypothesis that strains III, IV and V produce three different analogues of the Vfm QS signal which also differ from the analogue produced by strains I and II. This hypothesis is clearly in agreement with the experimental data which indicate that strains III and IV produce two different analogues also differing from those produced by strains I and II.

### The polymorphism in the biosynthesis gene *vfmW* is linked to the polymorphism in the biosynthesis gene *vfmP*

A polymorphism was also observed within the biosynthesis gene *vfmW* of the different strains (Fig. 3). The phylogenetic tree based on the alignment of the variable region of the gene *vfmW* from 18 strains indicates that *vfmW* sequences from strains IV and V cluster in two specific phyla compared to the *vfmW* sequences from strains I, II and III (Fig. 4D). Strains IV and V respectively possess the variants *vfmP3* and *vfmP4* which are not shared by the other Vfm genetic groups I, II and III. This observation suggests that the polymorphism of *vfmW* is associated with the polymorphism of *vfmP*. A suitable explanation would be that the amino acid incorporated by the enzyme VfmP into the Vfm QS signal could be the substrate of the designated oxoacyl‐ACP synthase VfmW. Thus, the polymorphism of the gene *vfmW* would result from an adaptation to variations of this amino acid. As the NRPS signatures associated with the variants *vfmP1* and *vfmP2* are proposed to encode the same amino acid, the substrate of VfmW could be identical for strains I, II and III. This could explain why the *vfmW* sequences of strains I, II and III cluster together but not with the *vfmW* sequences of strains IV or V (Fig. 4D). Surprisingly, the *vfmW* sequences of the *D*. *chrysanthemi* strains II and III seem to be distant from the other *vfmW* sequences of the groups II and III.

### Use of the biosensor strains strongly suggests that strains I, III and IV produce three different sensor/receptor proteins of the Vfm QS signal

The two biosensor strains of group I (I‐uidA and I‐luc) are activated only by samples prepared from strains I or II (expected to produce the same analogue AI/II) (Tables 2 and 3). The biosensor strain of group IV (IV‐luc) is specifically activated by samples prepared from strains IV (Table 3). These patterns of specificity demonstrate that the sensory systems involved in the recognition of the Vfm QS signals AI/II and AIV are highly specific of their cognate signal. In contrast, the biosensor strain of group III (III‐luc) gave a positive response with samples prepared from all strains III but also from several strains I, II, or V (Table 3). This low specificity indicates that the Vfm sensor/receptor system of group III is less selective than those of other groups and is compatible with several analogues of the Vfm QS signal. However, the low reproducibility observed between replicates using the biosensor III‐luc with samples prepared from six strains of group I, one strain of group II and one strain of group V (Table 3) suggests that the affinity of the sensor/receptor system of group III is lower for the analogues AI/II and AV than for the analogue AIII.

Altogether, these results show that the groups I/II, III and IV produce three different variants of the Vfm protein responsible for the recognition of the Vfm QS signal. The *vfm* gene cluster encodes the two‐component regulatory system VfmH/VfmI. By analogy with classical two‐component systems, the histidine kinase VfmI could have been the sensor/receptor of the Vfm QS signal. However, no *vfmI* polymorphism associated with those observed in the signal biosynthesis genes *vfmO/P* has been identified, as shown by the *vfmI* phylogenetic tree which correlates with trees of housekeeping genes but not with the trees of *vfmO* or *vfmP* (Fig. 4; Fig. S2). Since a polymorphism resulting from its adaptation to the different analogues is expected for the gene encoding the signal sensor/receptor, VfmI does not appear to be a suitable candidate for this function. Although the transcriptional regulator VfmH was experimentally shown to be involved in the transduction of the cellular response to the Vfm QS signal (Nasser *et al*., 2013), to date, the function of the histidine kinase VfmI has not been experimentally investigated.

### The polymorphism of the ABC permease gene *vfmG* suggests an adaptation of the membrane protein VfmG to the specific binding of different analogues of the Vfm signal

In contrast to the histidine kinase *vfmI* gene, the ABC permease gene *vfmG* shows a polymorphism associated with those of the genes *vfmO/P*, as the clustering of the *vfmG* sequences observed on the *vfmG* phylogenetic tree coincides with the Vfm genetic groups defined on the basis of the *vfmO/P* variants (Fig. 4C). According to these phylogenetic data, the polymorphism of the ABC permease protein VfmG could be linked to an adaptation of the protein VfmG to specifically bind the different analogues of the Vfm QS signal.

To support this hypothesis, we noticed that several ABC permeases have been described as acting as co‐sensors/receptors of two‐component regulatory systems (Piepenbreier *et al*., 2017). An interesting example is the ABC permease BceB which acts as an accessory sensor/receptor in the two‐component regulatory pathway controlling the antibiotic resistance system Bce of *Bacillus subtilis* (Koh *et al*., 2020). This example is of particular interest, as the histidine kinases of the Vfm and Bce systems both exhibit an atypical topographical organization of their functional domains. In contrast to classical histidine kinases, they both lack any predicted ligand‐binding domain, as their two N‐terminal transmembrane helices are separated by a very short loop (data not shown). The absence of a ligand‐binding domain in the histidine kinase VfmI would explain why the polymorphism in the gene *vfmI* is not genetically linked to the polymorphism in the signal biosynthesis genes *vfmO/P*. Thus, VfmG is a better candidate than VfmI for direct binding to the Vfm signal. However, additional experimental data will be needed to clarify the role of VfmG as sensor/receptor of the Vfm QS signal.

### The biological assays show that spontaneous null mutations in the hypothetical protein *vfmX* gene or in the multidrug transporter *vfmC* gene do not affect the functioning of the Vfm system

The fully sequenced genome of the wild type *D*. *dadantii* strain DSM 18020^T^ (CFBP 1269^T^, NCPPB 898^T^) contains a frameshift in the gene *vfmX* (Table S1) also observed in the draft genome available for this strain (contig AOOE01000040.1 in GenBank). Since this strain gave a positive response with the biosensors I‐uidA and I‐luc (Tables 2 and 3), this *vfmX* frameshift does not block the production or secretion of the Vfm QS signal. This is consistent with the previous observation that a *vfmX* engineered null mutant of *D*. *dadantii* 3937 is not affected for the Vfm QS activity ((Nasser *et al*., 2013), *vfmX* is annotated as ID16066 in this reference). The gene *vfmX*, encoding a protein of unknown function, could therefore only play a minor role in the Vfm QS system. Alternatively, its inactivation may be functionally complemented by another unidentified gene present in the *D. dadantii* genome.

Four *D*. *dianthicola* strains, including CFBP 2982, exhibit a 1‐kb deletion in the gene *vfmC* (Table S1). In addition, a defect in assembly of the *vfmC* gene is observed in five other *D*. *dianthicola* genomes (Table S1), suggesting that the *vfmC* gene is also inactivated in these strains. Since strain CFBP 2982 gave a positive response with both biosensors I‐uidA and I‐luc (Tables 2 and 3), the 1‐kb deletion in the gene *vfmC* does not prevent the production or secretion of the Vfm QS signal. The gene *vfmC*, encoding a multidrug transporter, was proposed to be involved in the secretion of the Vfm QS signal synthesized by the bacteria (Nasser *et al*., 2013). However, the inactivation of this gene may be functionally complemented by another multidrug transporter gene present in the *D*. *dianthicola* genome.

### The polymorphism in the *vfm* genes could have an impact on the pathogenicity and epidemiology of *Dickeya* strains

The analogy of the Vfm system with the Agr QS specificity groups, which are associated with variations in virulence in *S*. *aureus* (Geisinger *et al*., 2012), raise further questions on the impact of the allelic variations within the *vfm* genes on the phytopathogenicity of the *Dickeya* strains. Although there are, to date, no comparative studies on the virulence of *Dickeya* strains on the basis of their appurtenance to different Vfm genetic groups, we just noticed that almost all strains of the two economically important species *D*. *solani* and *D*. *oryzae* belong to the Vfm genetic group III (Fig. 2), suggesting that the Vfm QS system could be particularly efficient in this group. In contrast, all strains of *D*. *aquatica* or *D*. *lacustris*, two species not linked to agricultural damages but found in water, belong to group IV. Strains of the poorly represented species *D*. *poaceiphila*, found to be weakly virulent under laboratory conditions (Hugouvieux‐Cotte‐Pattat *et al*., 2020a), are the sole members of group V (Fig. 2). However, this species may have a quite restricted host range and niche as *D*. *poaceiphila* isolates were found only on plants of the *Poaceae* family (grasses) and only in Australia (Hugouvieux‐Cotte‐Pattat *et al*., 2020a).

A recent study proposed that an allelic variation in the hypothetical protein VfmB is associated with variations in the bacterial aggressiveness and competitiveness in *D*. *solani* (Blin *et al*., 2021). Four *D*. *solani* strains possessing the allele VfmBSer were shown to more efficiently damage potato tubers than four *D*. *solani* strains possessing the allele VfmBPro. However, this VfmB allelic variation is not linked to any variation in the specificity of the Vfm QS system since the eight *D*. *solani* strains tested share exactly the same proteins VfmG, VfmO, VfmP and VfmW (amino acid identity of 100%). As most *D*. *solani* strains, these eight strains belong to the genetic group III and positively respond to the biosensor III‐luc (Table 3).

Both predictive *in silico* analyses and experimental results demonstrated that different strains of the same *Dickeya* species could belong to different Vfm genetic groups (Fig. 2). Thus, signalling *via* the Vfm QS system is limited to strains belonging to compatible groups. This observation raises questions regarding the cohabitation inside the same host organism or the same environment of strains belonging to different Vfm genetic groups (Fig. 2): for example, the species *D*. *fangzhongdai* includes strains of groups I and III. In this species, strains of group I can act as cheaters when they live next to strains of group III because group III strains respond to the signalling molecule AI while strains of group I do not respond to the signalling molecule AIII. Regarding species containing strains of group III and IV, such as *D*. *chrysanthemi*, *D*. *fangzhongdai*, *D*. *parazeae* or *D*. *zeae*, cohabitation of strains unable to communicate via the Vfm QS system could occur since strains of group III do not respond to the analogue AIV and inversely strains IV do not respond to the analogue AIII. Similarly, the communication via Vfm is impossible between strains I and strains IV, for example in the species *D*. *dadantii*. On the contrary, the absence of *vfm* polymorphism was observed in a few *Dickeya* species. While only a limited number of strains have been sequenced for *D*. *aquatica*, *D*. *lacustris* or *D*. *poaceiphila*, *D*. *dianthicola* is a good example of species homogeneity as all 33 analyzed strains of this species belong to the Vfm genetic group I (Fig. 2, Table S1), as well as the 16 recently sequenced strains (data not shown, Curland *et al*., 2021).

## Conclusion

This study demonstrates the presence of polymorphism in a few genes of the *vfm* cluster of *Dickeya* strains and it provides strong evidence that the strain‐specific polymorphism observed in the biosynthesis genes *vfmO/P* results in the production of at least three different analogues of the Vfm QS signal. This genetic polymorphism has a direct consequence on the specificity of the Vfm QS system. Interestingly, strains belonging to a same *Dickeya* species could belong to different Vfm specificity groups, opening new questions that remain to be explored, for instance regarding the impact of the Vfm polymorphism on the pathogenicity and the epidemiology of *Dickeya* strains. Does the efficiency of the Vfm QS system vary according to the Vfm polymorphism? Is this strain polymorphism linked to variations in virulence? Do strains belonging to different Vfm specificity groups coexist naturally in a same niche?

The new information about the Vfm QS system evidenced by this study could be of great interest for developing quorum quenching techniques in order to disrupt communication between *Dickeya* cells, with the aim to reduce their virulence. The discovery of a strain‐specific polymorphism in the genes *vfmO/P* linked to variations in the specificity of production and recognition of different Vfm analogues is an essential knowledge for the development of control methods targeting the Vfm QS system of *Dickeya* strains.

## Experimental procedures

### Bacterial strains, media and culture conditions

The bacterial strains of different *Dickeya* species were obtained from international culture collections. All strains were grown at 30°C in rich medium LB or in minimal medium M63 (Miller, 1992) containing glycerol at a final concentration of 2 g L^−1^ as a carbon source. Kanamycin (Km) was used at a final concentration of 20–50 μg ml^−1^. Ampicillin was used at a final concentration of 20–50 μg ml^−1^. Chloramphenicol was used at a final concentration of 12.5 μg ml^−1^. For solid media, 15 g L^−1^ agar was usually added.

### Detection of the Vfm signal using the Vfm biosensor I‐uidA

The biosensor I‐uidA corresponds to the strain A5243 previously prepared from the model strain *D*. *dadantii* 3937 by introducing a *vfmE*‐*uidA* Km^R^ mutation (Nasser *et al*., 2013). The biosensor I‐uidA was used to test the cell‐free culture supernatants of 52 wild‐type strains belonging to different *Dickeya* species. The strains were grown in M63 medium supplemented with glycerol. After 24 h at 30°C, cells were discarded by centrifugation at 12 000*g* for 4 min and the culture supernatants were filtered and stored at 4°C. These supernatants were added in a 1/10 ratio to early exponential phases of A5243 cultures in M63 medium supplemented with glycerol and kanamycin. After 24 h at 30°C, the response of the reporter system was detected by a spectrophotometric assay of the *uidA* product. ß‐Glucuronidase (GUS) activity was measured by monitoring the hydrolysis of *p*‐nitrophenyl‐ß‐d‐glucuronide to yield *p*‐nitrophenol that absorbs at *λ* = 405 nm (Bardonnet and Blanco, 1992). Supernatants of the wild‐type strain 3937 and of the *vfmE* mutant A5243 were used as positive and negative controls respectively. A sample was considered as positive if its GUS activity was at least five times higher than that of the negative control.

### Construction of the Vfm biosensor I‐luc

The 5′ regulatory region of the *vfmE* gene of strain *D*. *dadantii* 3937 was amplified by PCR with the primers RRvfmEdeb XhoI (GGCTCGAGAGGTCGTTTCCTGTTCATCTGCGTC) and RRvfmEfin BamHI (GGGGATCCGTAGGTGTTCTGCAAGCTCATG). After treatment with both restriction enzymes XhoI and BamHI, the resulting PCR fragment was cloned between the restriction site XhoI and BglII of the plasmid pUCTer‐Luc‐Cm (Jiang *et al*., 2015). The resulting plasmid was introduced by electroporation in the *vfmE* mutant A5243 (Nasser *et al*., 2013).

### Construction of the Vfm biosensors III‐luc and IV‐luc

The biosensors III‐luc and IV‐luc were derived from strains *D*. *solani* IPO 2222 and RNS05‐1‐2A, respectively. They correspond to an in frame deletion of the *vfmE* coding sequence associated with its replacement by the luciferase gene *luc* (∆*vfmE‐luc* constructions). They were obtained using a technique involving a suicide plasmid and the *sacB* counter‐selection (Edwards *et al*., 1998). The vector pRE112, an R6K‐based suicide plasmid carrying the genes *sacB* and *cat* (Cm^R^), has previously been successfully used for allelic exchange in *D*. *dadantii* (Royet *et al*., 2019). The plasmid pRE112‐∆*vfmE‐luc* was constructed by cloning simultaneously three PCR fragments, consisting of two 1‐kbp DNA fragments corresponding to the upstream and downstream *vfmE* coding sequence of *D*. *solani* IPO2222 and the *luc* coding sequence, into the SacI/KpnI digested vector pRE112, using the Gibson's assembly method (Gibson *et al*., 2009). After CaCl_2_‐mediated transformation of *E*. *coli* DH5α λpir competent cells, transformants were selected onto LB‐Cm plates. Plasmids were extracted with the NucleoSpin Plasmid kit (Macherey‐Nagel, Düren, Germany), checked by restriction digestion and PCR analysis. They were transferred by transformation into competent cells of *E*. *coli* MFDpir, a strain producing the RP4 conjugation machinery which allows the transfer of plasmids into several Gram‐negative bacteria by conjugation (Ferrières *et al*., 2010). After mating of *E*. *coli* MFDpir with *D*. *solani* IPO 2222 or RNS05‐1‐2A, cells were spread onto LB‐Cm plates to select the first recombination of the pRE112‐∆*vfmE‐luc* plasmid into the *D*. *solani* chromosome. After a second isolation on this medium, colonies were spread onto LB plates supplemented with 5% sucrose and incubated at 20°C for 2–3 days to allow the second recombination event leaving the ∆*vfmE‐luc* constructions derived from *D*. *solani* IPO 2222 or RNS05‐1‐2A, respectively. Sucrose‐resistant colonies were replicated onto LB‐Cm plates to check plasmid loss. The correct structure of the *vfmE‐luc* fusion was confirmed by PCR analysis.

### Detection of the Vfm signal using the biosensors I‐luc, III‐luc and IV‐luc

A biological assay allowing the detection of the Vfm QS signal was carried out in 96‐well plates (Thermo Scientific Nunc, Rochester, NY, USA) displaying wells shaped with a flat transparent bottom to allow measurement of the optical density and a white opaque wall to allow measurement of bioluminescence without interference from the neighbour wells. As a standard procedure, 20 μl of the sample to be tested (a bacterial culture supernatant) was mixed in each well with 180 μl of a 5‐h culture in LB medium supplemented with kanamycin and ampicillin (for the biosensor I‐luc) or chloramphenicol (for the biosensors III‐luc and IV‐luc) and with Xenolight™ D‐luciferin (Perkin‐Elmer, Waltham, MA, USA) at a final concentration of 225 ng ml^−1^. Optical density at 600 nm and bioluminescence were concomitantly measured every 15 min for 15 h in a Tecan Spark plate reader (Tecan, Grödig, Austria). The internal temperature was set to 28°C and an orbital agitation (150 rpm, orbital radius 4 mm) was continuously applied to the plate between each measurement point. Results are expressed as the mean of the 61 bioluminescence measurements under the codename ‘mean61’. A sample was considered as positive if its ‘mean61’ is at least twice the ‘mean61’ of the negative control (a cell‐free supernatant of a culture of the *vfmE* mutant A5243).

### Verification of the species appurtenance by sequencing a *gapA* PCR product

For strain identification, the gene *gapA* was amplified by PCR performed using the Illustra™ PuReTaq™ Ready‐To‐Go™ kit (GE Healthcare, Chicago, IL, USA) on bacterial cell lysates with the primers gapAF and gapAR (Cigna *et al*., 2017). Sequences of the PCR products were determined by Sanger sequencing (Biofidal, Vaulx‐en‐Velin, France).

### Genome sequencing

The genomic DNA of each strain was extracted using a NucleoSpin^R^ bacterial DNA purification kit (Macherey‐Nagel). Illumina sequencing and assemblies of the reads were performed by MicrobesNG (Birmingham, UK). The resulting draft genome sequences were used to determine the sequence of the complete *vfm* locus of the strains *D*. *fangzhongdai* NCPPB 2929 (GenBank accession no. MZ611617), *D*. *chrysanthemi* CFBP 1270 (GenBank accession no. MZ611618), *D*. *dadantii* NCPPB 3065 (GenBank accession no. MZ611619) and *D*. *dadantii* CFBP 3694 (GenBank accession no. MZ611620).

### Comparative analysis of the genomic sequences available in GenBank

The sequence of the *vfm* locus of 126 genomes of *Dickeya* available until April 2021 was retrieved from GenBank. The type I NRPS‐like signature of the proteins VfmM, VfmO and VfmP was determined with NRPSpredictor2 (Röttig *et al*., 2011). Amino acid identities between the different allelic forms of the proteins VfmO, VfmP, VfmG and VfmW were determined with Clustal Omega (Sievers *et al*., 2011). Alignment of the nucleotide sequences of the *vfm* loci from different pairs of strains of *Dickeya* was performed with Nucleotide BLAST (Basic Local Alignment Search Tool) or Clustal Omega (Sievers *et al*., 2011). Transmembrane helices within the proteins VfmG and VfmI were predicted with HMMTOP (Tusnády and Simon, 2001). Conserved domains within the proteins VfmF, VfmG, VfmH and VfmI have been searched with the CDD/SPARCLE database (Lu *et al*., 2020). Phylogenetic trees were generated with a ready‐to‐use pipeline [(Dereeper *et al*., 2008), Phylogeny.lirmm.fr]. Sequences were aligned with MUSCLE (Edgar, 2004), and each phylogenetic tree was reconstructed using the maximum likelihood method implemented in the PhyML program (Guindon and Gascuel, 2003). The graphical representation of the phylogenetic tree was performed with TreeDyn (Chevenet *et al*., 2006).

## Supporting information


**Appendix** **S1**. Supporting Information.

**Fig. S1.** Alignment of the sequence located between the core domains A4 and A5 in the different variants of the proteins VfmP1 to VfmP4 and VfmO1 to VfmO3.

**Fig. S2.** Phylogenetic trees based on the genes *gapA* and *vfmI* of a selection of *Dickeya* strains (same selection as for Fig. 4).

**Table S1.** Complete data on the *vfm* gene cluster of the 126 *Dickeya* strains whose genomes are available until April 2021.
