# Supplementary material for: Specificity and genetic polymorphism in the Vfm quorum sensing system of plant pathogenic bacteria of the genus Dickeya
Source: Environ Microbiol. 2022 Jan 10;24(3):1467–83. doi: 10.1111/1462-2920.15889 (PMC9306890; doi:10.1111/1462-2920.15889)
Supplement: Supplementary file 1 — Appendix S1. Supporting Information. [file EMI-24-1467-s001.docx]

The predicted transmembrane helices are highlighted in yellow.

dadantii3937 (group I) MNLWFDIRYALRLLLKSPGFSVLTITVMACGLGLALYMYSVINTIMYKTLPYPKGEGMVM

dianthicolaRNS04.9 (group I) MNVWFDIRYALRLLLKSPGFSVLTITVMACGLGLALYMYSVINTIMYKTLPYPKGEGMVM

solaniIPO2222 (group III) MNLWFDIRYALRLLLKSPGFSVLTITVMACGLGLALYMYSVINTIMYKTLPYPKGEGMVM

zeaeEC1 (group III) MNFWFDIRYALRLLLKSPGFSVLTITVMACGLGLALYMYSVINTIMYKTLPYPRGEGMVM

dadantii3537 (group IV) MNLWFDIRYALRLLLKSPGFSVLTITVMACGLGLALYMYSVINTIMYKTLPYPKGEGMVM

solaniRNS05.1.2A (group IV) MNLWFDIRYALRLLLKSPGFSVLTITVMACGLGLALYMYSVINTIMYKTLPYPKGEGMVM

**.**************************************************.******

dadantii3937 (group I) VTPNVGGVSLDDSGLNFLDYTELSRQSTKLDDIGYFYAEFLDLNDGNRSVKYIGILSTPH

dianthicolaRNS04.9 (group I) VTPNVGGVSLDDSGLNFLDYTELSRQSTKLDDIGYFYAEFLDLNDGNRSVKYIGILSTPH

solaniIPO2222 (group III) VTPNVGGVSLDDSGLNFLDYTELSRQLTKIDDVGYFYAEYLDLNDGKRSVRYIGILSTPQ

zeaeEC1 (group III) VTPNVGGVSLDDSGLNFLDYTELSRQLTKINDIGYFYAEYLDLNDGKNSARYIGILSTPQ

dadantii3537 (group IV) VTPKMDGEGLNDSGLNFLDYELIRQKATKLTDISYFYAEYADLKDDEKSVRYIAVYNTPD

solaniRNS05.1.2A (group IV) VTPKMDGEGLNDSGLNFLDYELIRQKATKLTDISYFYAEYADLKDDEKSVRYIAVYNTPD

***.:.* *:********* : :: *** *:.*****: **:* . *.:**.: .**

dadantii3937 (group I) IFSYTGTSPLMGRVLNDKDMQPDALPVTVISYDLWKSYFNGREDILKQSVQINGIRTYIV

dianthicolaRNS04.9 (group I) MFSYTGTSPLMGRVLNDKDMQPDALPVTVISYDLWKSYFNGREDILKQSVQINGVRTYIV

solaniIPO2222 (group III) MFDYTGVSPLIGRVLNDSDMLSGSVPVTVISYDLWENYFNGRKDILNQSVQINGVRTYIV

zeaeEC1 (group III) MFSYTGVSPLMGRIFNENDMLSGALPVTVISYDLWQNYFNGRNDILSQSVQINGIRTHIV

dadantii3537 (group IV) MFTYTGVSPFLGRAFNQQDMQPGAEPVAVISYLLWQSYFNGRSDILNQTIKVNGSNTRVV

solaniRNS05.1.2A (group IV) MFAYTGVSPFLGRAFNQQDMQPGAEPVAVISYLLWQSYFNGRSDILNQTVKVNGSNTRVV

:* ***.**::** :*:.** .: **:**** **:.***** *** *::::** .* :*

dadantii3937 (group I) GVMPKGFAFPFFHDIWLPSRIKPLAFSLRSQAPDVYVYAHLNPHFSLDEANQEIVSVMDK

dianthicolaRNS04.9 (group I) GVMPKGFAFPFFHDIWLPSRIKPLAFSLRSQAPDVYVYAHLNPHFSLDEANQEIVSVMDK

solaniIPO2222 (group III) GVMPKGFAFPFFHDLWLPSKIKLQSFSLRNEAPEVYVYAHLNPRFSLDEANQEIASVMER

zeaeEC1 (group III) GVMPKGFEFPFFHDLWLPSKIEQQSFSLRSQAPEVYVYAHVNPRYSLDEANQEIASVMKR

dadantii3537 (group IV) GVMPKGFAFPFYHDLWLPSRLDPKLFSDRELAPEVFVFGRLPKGGNTTAASHELDGLMQE

solaniRNS05.1.2A (group IV) GVMPKGFAFPFYHDLWLPSRLDPKLFPDRELAPEVFVFGRLPKGVNTAAASHELDGLMQE

******* ***:**:**** : * *. **:*:*:.:: . *.:*: .:*

dadantii3937 (group I) LADKYPESNKGVSAVALSFQVSFMGDDTAQVFLITLSAVAFVLLLACCNVGNLLLARSHQ

dianthicolaRNS04.9 (group I) LADKYPESNKGVSAVALSFQVSFMGDDTAQVFLITLSAVAFVLLLACCNVGNLLLARSHQ

solaniIPO2222 (group III) LADKYPESNKGISAIALSFQVSFMGDDTAQVFLITLSAVAFVLLLACCNVGNLLLSRSHQ

zeaeEC1 (group III) LADKYPESNKGVSAIALSFQVSFMGDDTAQVFLITLSAVAFVLLLACCNVGNLLLARSHQ

dadantii3537 (group IV) VARQYPKVNKGISANVLSFQENFTGEETAQTFFVMLSAVGFILLLACFNVGNLLLARSNQ

solaniRNS05.1.2A (group IV) VARQYPKVNKGISANVLSFQENFTGEETAQTFFVMLSAVGFILLLACFNVGNLLLARSNQ

:* :**: ***:** .**** .* *::*** *:: ****.*:***** *******:**:*

dadantii3937 (group I) RAREIAIRAALGSPMMRLVMQMLWESLIICILAGIVGVLLAAWGLDLTNKIFPRFVPTRV

dianthicolaRNS04.9 (group I) RAREIAIRAALGSPMMRLVMQMLWESLIICILAGIVGVLLAAWGLDLTNKIFPRFVPTRV

solaniIPO2222 (group III) RTREIAVRVALGSPMIRLVMQMLWESLIICILAGIVGVLLAAWGLDLTNTIFPRFVPNRV

zeaeEC1 (group III) RTREIAVRVALGSPMMRLIMQMLWESLIICILAGIVGVLLAAWGLDLTNTIFPRFVPNRV

dadantii3537 (group IV) RTREIAIRMALGSPTSRLVMQMLWESLIISSIAGVIGVLLASWGLDITNYIFPRFVPNKV

solaniRNS05.1.2A (group IV) RTREIAIRMALGSPTSRLVMQMLWESLIISSIAGVIGVLLASWGLDITNYIFPRFVPNKV

*:****:* ***** **:**********. :**::*****:****:** *******.:*

dadantii3937 (group I) PSWWHLSLDSSMIINAGVLVIVTAFITGALPAWKIANGQFFQALRDGTRGIPRYRTSKAG

dianthicolaRNS04.9 (group I) PSWWHLSLDSSMIVNASILVIVTAFITGALPAWKIANGQFFQALKDGTRGITRYRTSKAG

solaniIPO2222 (group III) PSWWHLSLDGSMILNAGILVIVTAFITGALPAWKIANGQFFQALRDGSRGIPGYRTSKAG

zeaeEC1 (group III) PSWWHLSLDANMIFNASVLVVVTAFITGALPAWKIANGQFFQALRDGARGIPGYRTSKAG

dadantii3537 (group IV) PVWWHLSLDGSVILDAVILILVTSLITSALPAWKIANGKFAYALRDGVNSDQGRKTGRFS

solaniRNS05.1.2A (group IV) PVWWHLSLDGSVILDAIVLILATSLITSALPAWKIANGKFAYALRDGVNSDQGRKTGRFS

* *******..:*.:* :*:: *::**.**********:* **:** .. :*.: .

dadantii3937 (group I) RSLVIVEMALSFSILCISILFLILVTKAKNVDYGVATDGYIISRVNLNKDSYSTAQSRRL

dianthicolaRNS04.9 (group I) RSLVIVEMALSFSILCISILFLILVTKAKNVDYGVATDGYLISRVNLNKDSYSTEQSRRS

solaniIPO2222 (group III) RSLVIIEVALSFSILCISILLLILVTKAKNADYGVATDGYLISRVNLNKDVYPNVQSRRD

zeaeEC1 (group III) RSLVIIEVALSFSILCISILLLILVTKAKNADYGVATDGYQISRLNLNKDVYPTPQSRRD

dadantii3537 (group IV) RTLVVIEVALSCSILCISVLLLFLVMRATKADYGVPIDHFLVAKINVDQDAYPDDDSRRK

solaniRNS05.1.2A (group IV) RTLVVIEVALSCSILCISVLLLFLVMRATKADYGVPIDHFLVAKINVDQDSYQDDDSRRK

*:**::*:*** ******:*:*:** :*.:.****. * : ::::*:.:* * .***

dadantii3937 (group I) FYLALSKQLHDIPNLTSVALTTSAPSEFTEAHQLLIENREDGVIGDEGYPMVNDVGVMPG

dianthicolaRNS04.9 (group I) FYLALSKQLHDIPNLTSEALTTSAPGEFTEAHQFLIENREDGVIGDEGYPMANDVGVMPG

solaniIPO2222 (group III) FYLELSKQLHDIPGITAESLTTSAPGEFTAAHQLLIENREDGSNGDEHYPMVNDVQVMPG

zeaeEC1 (group III) FYLSLSRQLQAIPDVAAESITTSAPGEFTAAHQLIIENREDGKNSDESDPMVNDVQVMPG

dadantii3537 (group IV) LYLKLLDQVGTIPGVQASGLTSSAPGQFTFPHQVVTENMEQDGQDALSYSLVNDVRVMPG

solaniRNS05.1.2A (group IV) LYLKLLDQVGTIPGVQASGLTSSAPGQFTFPHQVVTENMEQDGQDALSYSLVNDVRMMPG

:** * *: **.: . .:*:***.:** .**. ** *.. .:.*** :***

dadantii3937 (group I) SLTGMGVNVLYGREFSAQDNEKSLPVAVISESLARKYWPDGKSAIGKRIRFRNEDNTDWY

dianthicolaRNS04.9 (group I) SLTGMGVNVLYGREFSAQDNEKSLPVAVISESLARKYWPDGKSAIGKRIRFRSEDNTDWY

solaniIPO2222 (group III) GLAGMGVKVIYGREFSALDNEKSPPAAVISESLARKYWPDGKSAIGKRVRFRNEEDSGWY

zeaeEC1 (group III) SLIGMGATIIYGREFSAADNEKSTPVAVISESLAKKYWSDEKSAIGKRIRFRNEEESGWY

dadantii3537 (group IV) SLTAMGVKLLNGREFADGDTDATLPVAVVSESFIKKYWPHEKTVIGKRLRFRDGNDYRWF

solaniRNS05.1.2A (group IV) SLTAMGVKLLNGREFADGDTDATLPVAVVSDSFIKKYWPHEKTVIGKRLRFRDGNDYRWF

.* **. :: ****: * . : *.**:*:*: :***. *:.****:***. :: *:

dadantii3937 (group I) TVIGVVSHVIHGRPFSAFKNRATVYRSLLQR--PAPMVTIMLKSEHPVVLPEILKLALYN

dianthicolaRNS04.9 (group I) TVIGVVSHVIHGRPFSAFKNRATIYRSLLQR--PAPMVTIMLKSEHLVVLPEILKLALYN

solaniIPO2222 (group III) TIIGVVTHVVHGRPFSAFKNRSTVYRSLLQR--PTPMMTIMLKAEHLSALPEKLKIALYN

zeaeEC1 (group III) TIIGVTTHIVHGRPFSAFKNRATVYRSLLQR--PTPIMTIMLKSGRPSMLPERLKLALYN

dadantii3537 (group IV) TVVGVVSHIIHGRPFSEFKTRPTVYRSLMQLRQANSSLTIMLRAPQPQRFSKPLFNVLNE

solaniRNS05.1.2A (group IV) TVVGVVSHIIHGRPFSEFKTRPTVYRSLMQLRQVNPSLTIMLRAPQPQRLSKPLFNVLNE

*::**..*::****** **.*.*:****:* . :****:: : .. : .* :

dadantii3937 (group I) VDNLMPVGPPQALNDMIERNTFGVSVLANLFMLFGVITIVLSSSGIYAVTQNAISQRIQE

dianthicolaRNS04.9 (group I) VDNLMPVGPPQALNDMIERNTFGVSVLANLFMLFGVITIVLSSSGIYAVTQNAISQRIQE

solaniIPO2222 (group III) VDSMMPVAPPQTLNNMLERNTLGVRFVANLFMLFGIIAIVLSSSGIYAVTQNAISQRTQE

zeaeEC1 (group III) VDSMMPVATPQTLNNMLEKNTLGVRFVANLFMLFGVIAIVLSSSGIYAVTQNAISQRTQE

dadantii3537 (group IV) LGPAIESSQPQTLSDQLTRNTVGVQFVTNLFLLFGAAAMVLAASGIYGVTQHAINQRTQE

solaniRNS05.1.2A (group IV) LGPAIESTQPQTLSDQLTRNTVGVQFVTNLFLLFGAAAMVLAASGIYGVTQHAINQRTQE

:. : ** *.: : :** ** .::***:*** ::**::****.***:**.** **

dadantii3937 (group I) IGIRQVLGATPNHLLRMLMLQGVNQLIAGLILGLPLALLAAPRINRILGDGRIHFTLLFA

dianthicolaRNS04.9 (group I) IGIRQVLGATPNHLLRMLMLQGVNQLIAGLILGLPLALLAAPRINRILGDGRIHFTLLFA

solaniIPO2222 (group III) IGIRQVLGATPSHLLKMLMLQGVNQLIAGLILGLPLALLAAPGINRALGDGRGHFVLLFV

zeaeEC1 (group III) IGIRQVLGATPKHLLKMLMLQGANQLILGLILGLPLALFAAPGINRALGDGRGNFVLLFV

dadantii3537 (group IV) IGIRQALGATPTRLLRMLMFSGLRQLFAGLAVGLPLAIFAAPKINRVYGDGGGGFMLLFG

solaniRNS05.1.2A (group IV) IGIRQALGATPTRLLRMLMFSGLRQLFAGLALGLPLAIFAAPKINRVYGDGGGGFMLLFG

*****.*****..**:***:.* .**: ** :*****::*** *** *** * ***

dadantii3937 (group I) FVALFIVIIVALATWIPSRRVIMMKPGDAIRYE

dianthicolaRNS04.9 (group I) CVALFIVIIVALATWIPSRRVIMMKPGDAIRYE

solaniIPO2222 (group III) CVALFLMLIVALATWIPSRRVIMMKPGDAIRYE

zeaeEC1 (group III) CVALFIMTIVALATWIPSRRVIMMKPGDAIRYE

dadantii3537 (group IV) GVALFIVIIVALATWIPSRRVIMMKPGDAIRYE

solaniRNS05.1.2A (group IV) GVALFIVIIVALATWIPSRRVIMMKPGDAIRYE

*****: ************************
