# Supplementary material for: Specificity and genetic polymorphism in the Vfm quorum sensing system of plant pathogenic bacteria of the genus Dickeya
Source: Environ Microbiol. 2022 Jan 10;24(3):1467–83. doi: 10.1111/1462-2920.15889 (PMC9306890; doi:10.1111/1462-2920.15889)
Supplement: Supplementary file 2 — Fig. S1. Alignment of the sequence located between the core domains A4 and A5 in the different variants of the proteins VfmP1 to VfmP4 and VfmO1 to VfmO3. [file EMI-24-1467-s002.pptx]

## Slide 1
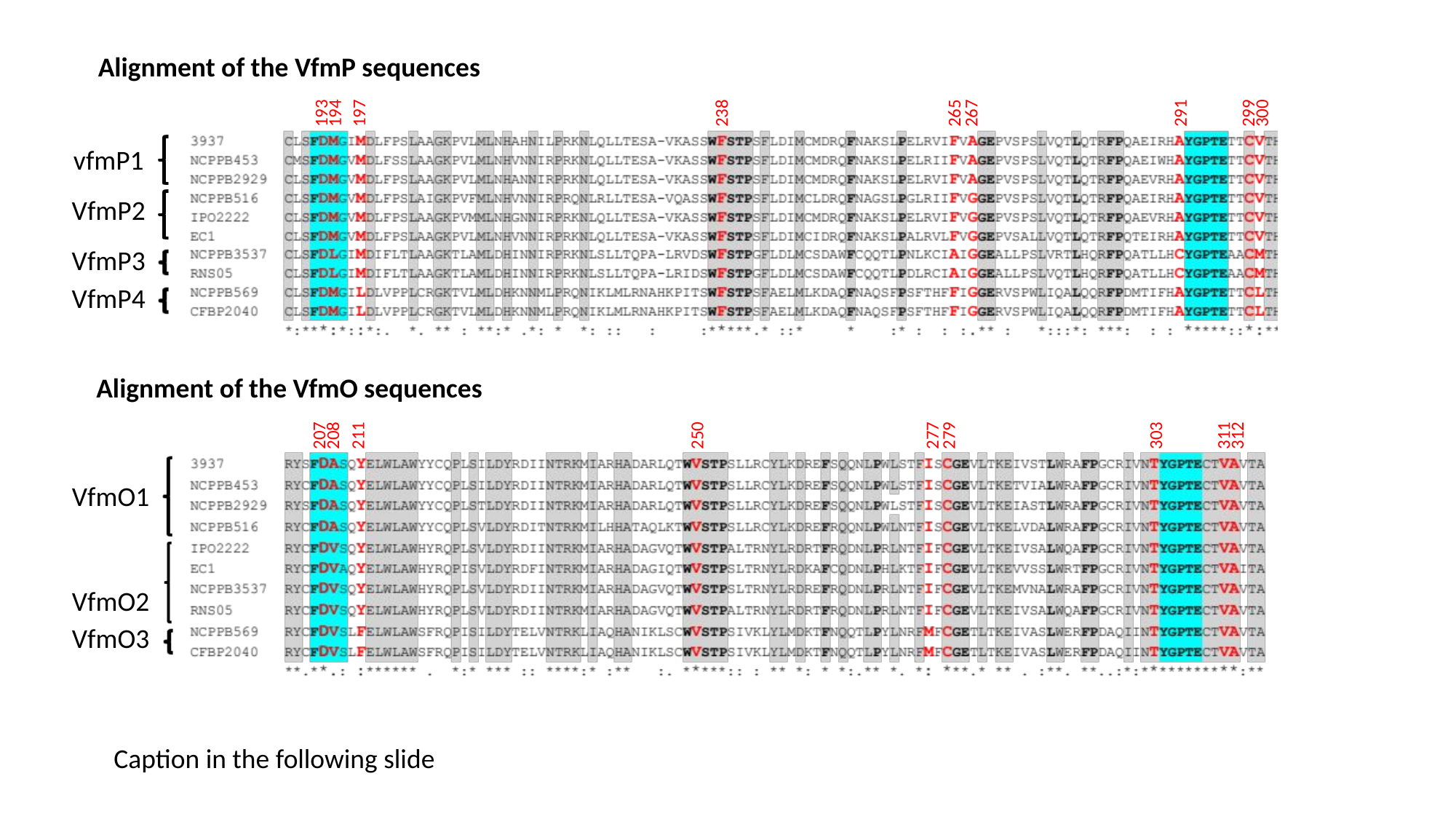

Alignment of the VfmP sequences
193
194
197
238
265
267
291
299
300
vfmP1
VfmP2
VfmP3
VfmP4
Alignment of the VfmO sequences
207
208
211
250
277
279
303
311
312
VfmO1
VfmO2
VfmO3
Caption in the following slide

## Slide 2
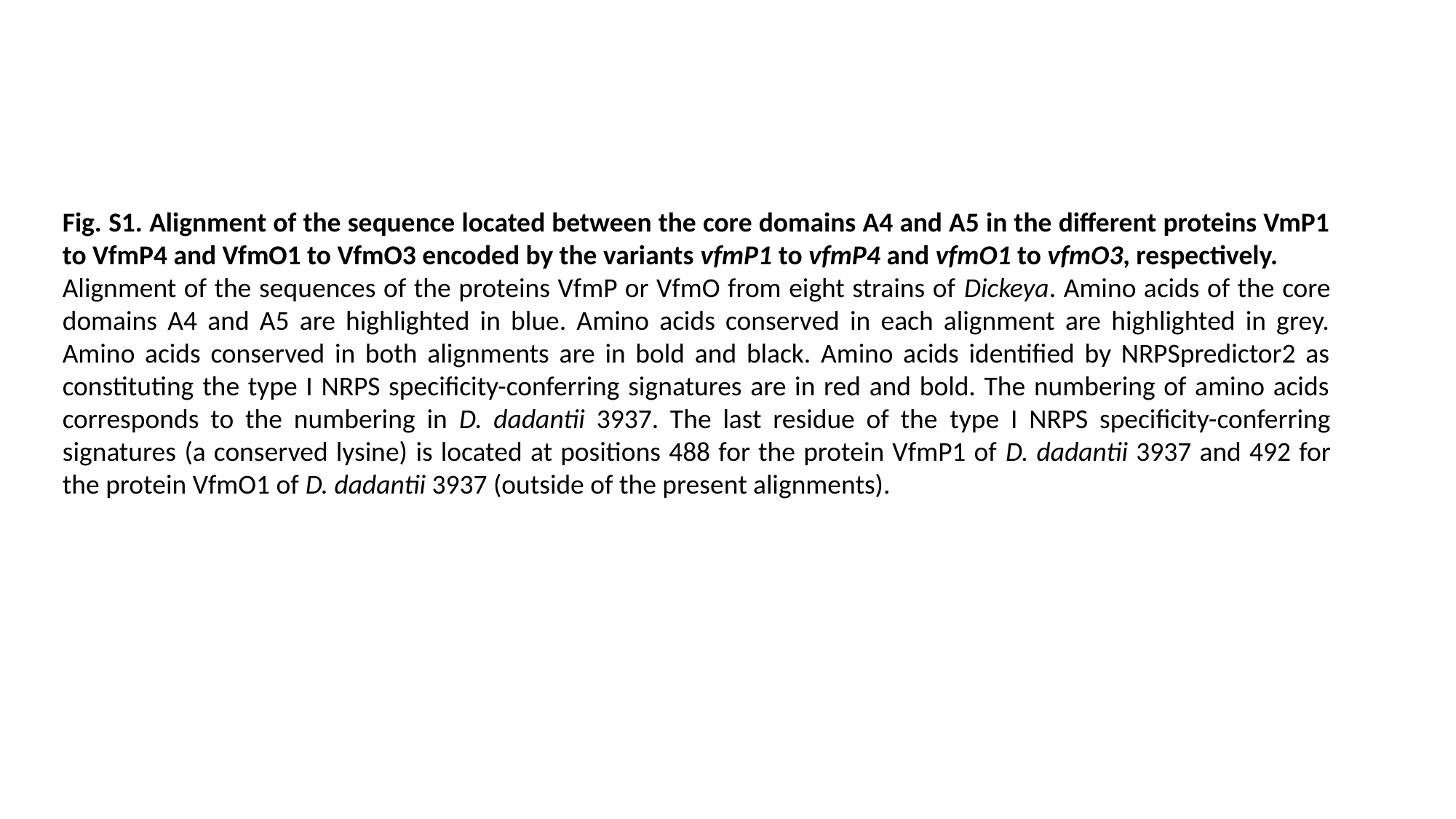

Fig. S1. Alignment of the sequence located between the core domains A4 and A5 in the different proteins VmP1 to VfmP4 and VfmO1 to VfmO3 encoded by the variants vfmP1 to vfmP4 and vfmO1 to vfmO3, respectively.
Alignment of the sequences of the proteins VfmP or VfmO from eight strains of Dickeya. Amino acids of the core domains A4 and A5 are highlighted in blue. Amino acids conserved in each alignment are highlighted in grey. Amino acids conserved in both alignments are in bold and black. Amino acids identified by NRPSpredictor2 as constituting the type I NRPS specificity-conferring signatures are in red and bold. The numbering of amino acids corresponds to the numbering in D. dadantii 3937. The last residue of the type I NRPS specificity-conferring signatures (a conserved lysine) is located at positions 488 for the protein VfmP1 of D. dadantii 3937 and 492 for the protein VfmO1 of D. dadantii 3937 (outside of the present alignments).
