# Supplementary material for: Specificity and genetic polymorphism in the Vfm quorum sensing system of plant pathogenic bacteria of the genus Dickeya
Source: Environ Microbiol. 2022 Jan 10;24(3):1467–83. doi: 10.1111/1462-2920.15889 (PMC9306890; doi:10.1111/1462-2920.15889)
Supplement: Supplementary file 3 — Fig. S2. Phylogenetic trees based on the genes gapA and vfmI of a selection of Dickeya strains (same selection as for Fig. 4). [file EMI-24-1467-s004.pptx]

## Slide 1
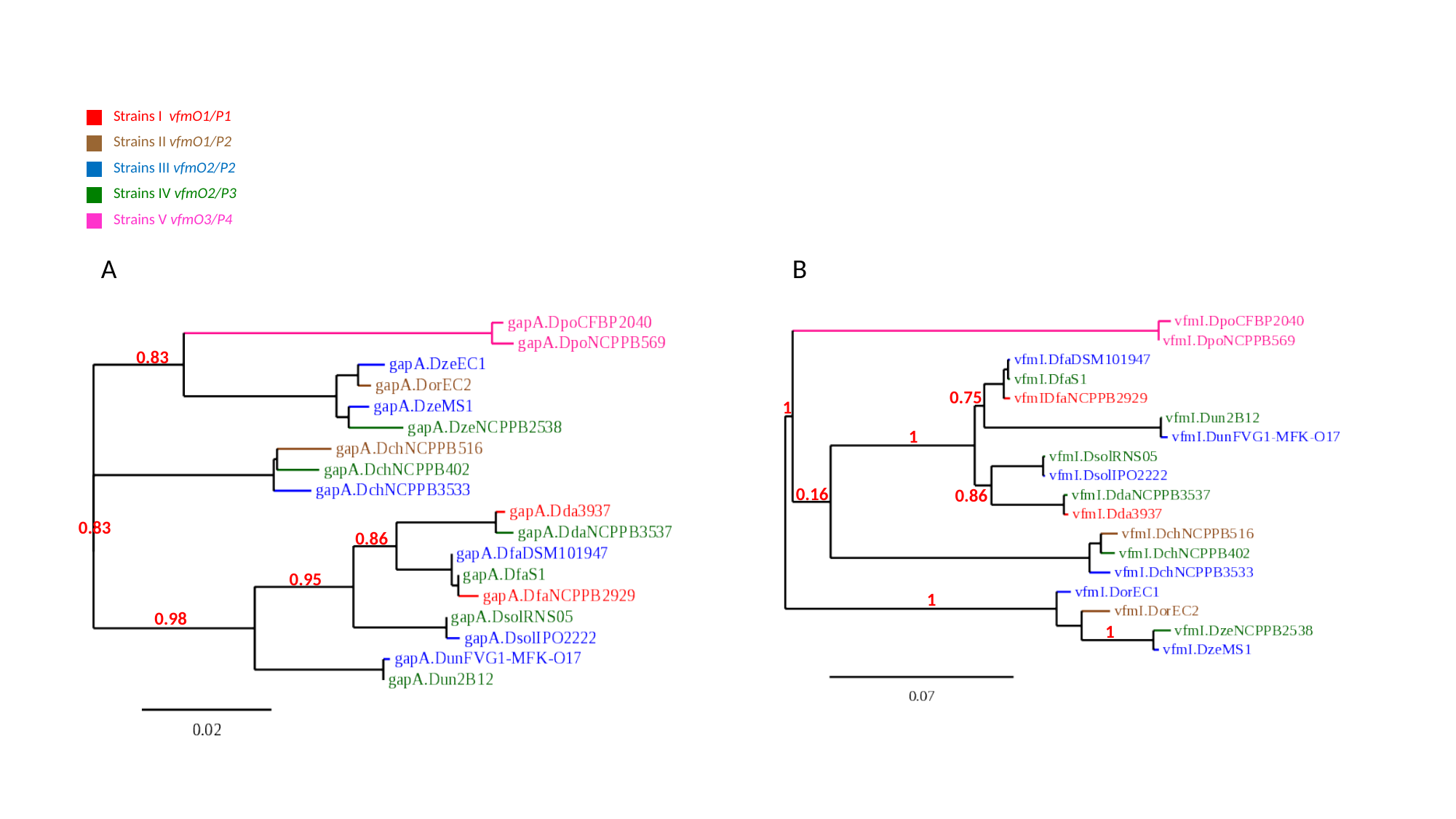

Strains I vfmO1/P1
Strains II vfmO1/P2
Strains III vfmO2/P2
Strains IV vfmO2/P3
Strains V vfmO3/P4
A
B
0.83
0.75
1
1
0.16
0.86
0.83
0.86
0.95
1
0.98
1

## Slide 2
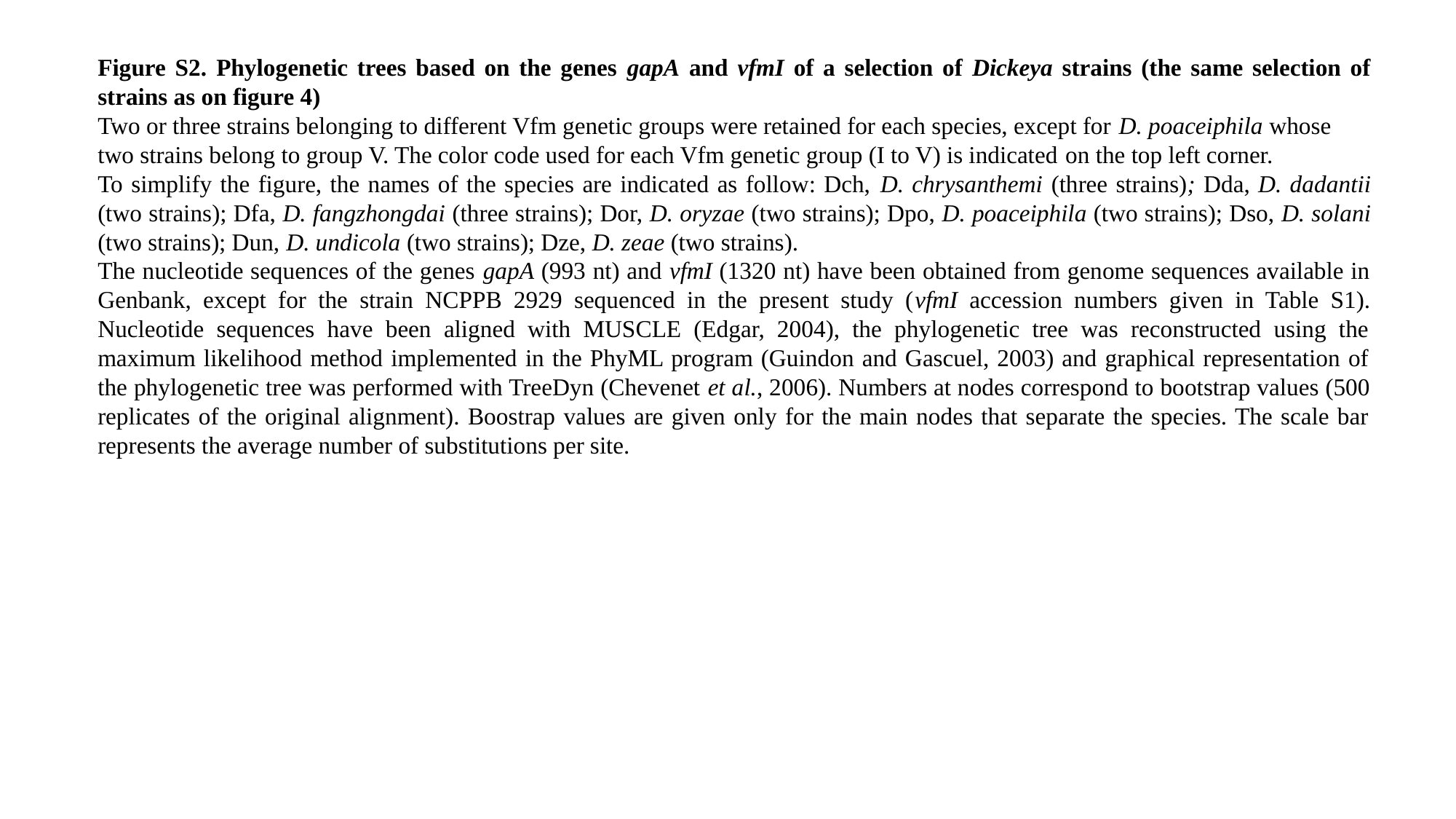

Figure S2. Phylogenetic trees based on the genes gapA and vfmI of a selection of Dickeya strains (the same selection of strains as on figure 4)
Two or three strains belonging to different Vfm genetic groups were retained for each species, except for D. poaceiphila whose two strains belong to group V. The color code used for each Vfm genetic group (I to V) is indicated on the top left corner.
To simplify the figure, the names of the species are indicated as follow: Dch, D. chrysanthemi (three strains); Dda, D. dadantii (two strains); Dfa, D. fangzhongdai (three strains); Dor, D. oryzae (two strains); Dpo, D. poaceiphila (two strains); Dso, D. solani (two strains); Dun, D. undicola (two strains); Dze, D. zeae (two strains).
The nucleotide sequences of the genes gapA (993 nt) and vfmI (1320 nt) have been obtained from genome sequences available in Genbank, except for the strain NCPPB 2929 sequenced in the present study (vfmI accession numbers given in Table S1). Nucleotide sequences have been aligned with MUSCLE (Edgar, 2004), the phylogenetic tree was reconstructed using the maximum likelihood method implemented in the PhyML program (Guindon and Gascuel, 2003) and graphical representation of the phylogenetic tree was performed with TreeDyn (Chevenet et al., 2006). Numbers at nodes correspond to bootstrap values (500 replicates of the original alignment). Boostrap values are given only for the main nodes that separate the species. The scale bar represents the average number of substitutions per site.
